# Metabolic Regulation in Progression to Autoimmune Diabetes

**DOI:** 10.1371/journal.pcbi.1002257

**Published:** 2011-10-27

**Authors:** Marko Sysi-Aho, Andrey Ermolov, Peddinti V. Gopalacharyulu, Abhishek Tripathi, Tuulikki Seppänen-Laakso, Johanna Maukonen, Ismo Mattila, Suvi T. Ruohonen, Laura Vähätalo, Laxman Yetukuri, Taina Härkönen, Erno Lindfors, Janne Nikkilä, Jorma Ilonen, Olli Simell, Maria Saarela, Mikael Knip, Samuel Kaski, Eriika Savontaus, Matej Orešič

**Affiliations:** 1 VTT Technical Research Centre of Finland, Espoo, Finland; 2 Aalto University School of Science, Department of Information and Computer Science, Helsinki Institute for Information Technology HIIT, Espoo, Finland; 3 Helsinki Institute for Information Technology HIIT, Department of Computer Science, University of Helsinki, Finland; 4 Department of Pharmacology, Drug Development and Therapeutics, University of Turku, Turku, Finland; 5 Hospital for Children and Adolescents, University of Helsinki, Helsinki, Finland; 6 Finnish Red Cross Blood Service, Helsinki, Finland; 7 Department of Clinical Microbiology, University of Eastern Finland, Kuopio, Finland; 8 Immunogenetics Laboratory, University of Turku, Turku, Finland; 9 Department of Pediatrics, University of Turku, Turku, Finland; 10 Department of Pediatrics, Tampere University Hospital, Tampere, Finland; 11 Folkhälsan Research Center, Helsinki, Finland; 12 Unit of Clinical Pharmacology, Turku University Hospital, Turku, Finland; 13 Institute of Molecular Medicine Finland FIMM, Helsinki, Finland; University of Wisconsin-Madison, United States of America

## Abstract

Recent evidence from serum metabolomics indicates that specific metabolic disturbances precede β-cell autoimmunity in humans and can be used to identify those children who subsequently progress to type 1 diabetes. The mechanisms behind these disturbances are unknown. Here we show the specificity of the pre-autoimmune metabolic changes, as indicated by their conservation in a murine model of type 1 diabetes. We performed a study in non-obese prediabetic (NOD) mice which recapitulated the design of the human study and derived the metabolic states from longitudinal lipidomics data. We show that female NOD mice who later progress to autoimmune diabetes exhibit the same lipidomic pattern as prediabetic children. These metabolic changes are accompanied by enhanced glucose-stimulated insulin secretion, normoglycemia, upregulation of insulinotropic amino acids in islets, elevated plasma leptin and adiponectin, and diminished gut microbial diversity of the *Clostridium leptum* group. Together, the findings indicate that autoimmune diabetes is preceded by a state of increased metabolic demands on the islets resulting in elevated insulin secretion and suggest alternative metabolic related pathways as therapeutic targets to prevent diabetes.

## Author Summary

We have recently found that distinct metabolic disturbances precede β-cell autoimmunity in children who later progress to type 1 diabetes (T1D). Here we performed a murine study using non-obese diabetic (NOD) mice that recapitulated the protocol used in human, followed up by independent studies where NOD mice were studied in relation to risk of diabetes progression. We found that young female NOD mice who later progress to autoimmune diabetes exhibit the same lipidomic pattern as prediabetic children. These metabolic changes are accompanied by enhanced glucose-stimulated insulin secretion, upregulation of insulinotropic amino acids in islets, elevated plasma leptin and adiponectin, and diminished gut microbial diversity of the *Clostridium leptum* subgroup. The metabolic phenotypes observed in our study could be relevant as end points for studies investigating T1D pathogenesis and/or responses to interventions. By proceeding from a clinical study *via* metabolomics and modeling to an experimental model using a similar study design, then evolving further to tissue-specific studies, we hereby also present a conceptually novel approach to reversed translation that may be useful in future therapeutic studies in the context of prevention and treatment of T1D as well as of other diseases characterized by long prodromal periods.

## Introduction

Type 1 diabetes (T1D) is an autoimmune disease that results from the selective destruction of insulin-producing β-cells in pancreatic islets. The diagnosis of T1D is commonly preceded by a long prodromal period which includes seroconversion to islet autoantibody positivity [1] and subtle metabolic disturbances [2]. The incidence of T1D among children and adolescents has increased markedly in the Western countries during the recent decades [3] and is presently increasing at a faster rate than ever before [4], [5]. This suggests an important role of environment and gene-environment interactions in T1D pathogenesis.

Metabolome is sensitive to both genetic and early environmental factors influencing later susceptibility to chronic diseases [6]. Recent evidence from serum metabolomics suggests that metabolic disturbances precede early β-cell autoimmunity markers in children who subsequently progress to T1D [2]. However, the environmental causes and tissue-specific mechanisms leading to these disturbances are unknown. Given its relatively low disease incidence in the general population and even among subjects at genetic risk [1], studies on early phenomena of T1D pathogenesis in humans are a huge undertaking as they require long and frequent follow-up of large numbers of subjects [2], [7], [8] to be able to go “back to the origins” of the disease once a sufficient number of subjects in the follow-up have progressed to overt disease. In order to effectively prevent this disease it is thus fundamental to identify suitable experimental models that recapitulate findings from such large-scale clinical studies while being amenable to mechanistic studies at the systems level.

The non-obese diabetic (NOD) mouse is a well characterized model of autoimmune disease [9] which has been widely used in studies of T1D. It is clear that the NOD experimental model does not completely mimic the immune system and T1D pathogenesis in man [10]. Only a fraction of NOD mice progress to disease, with the incidence of spontaneous diabetes being 60%–80% in females and 20%–30% in males [9]. There is thus a stochastic component to diabetes pathogenesis in NOD mice, believed to be due to random generation of islet-specific T cells [11]. The disease incidence does seem to depend on the environment and there is evidence indicating that it is the highest in a relatively germ-free environment [12] and that gut microbiota may affect disease incidence *via* the modulation of the host innate immune system [13].

**Figure 1:**
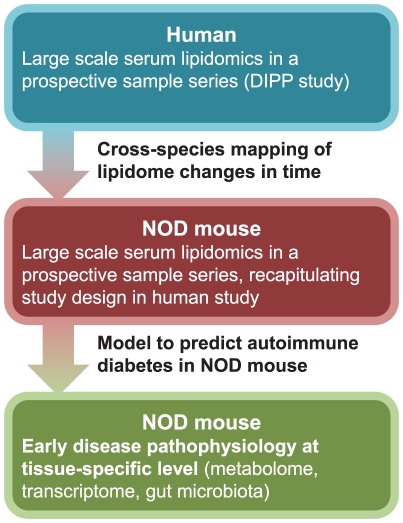
Reverse-translational setting of the study. Starting from clinical observations using metabolomics [2], then proceeding *via* modeling and metabolomics to an experimental model using a similar study design, then evolving further to tissue-specific studies. Such an approach aims to facilitate studies of early prodromal phases of disease pathogenesis.

Herein we performed a murine study in NOD mice that recapitulated the protocol used in human studies [2] and applied a reverse-translational approach (Figure 1) to (1) map the lipidomic profiles of T1D progressors in human studies to lipidomic profiles in NOD mice and derive a surrogate marker to stratify mice according to risk of developing autoimmune diabetes, then (2) perform multiple follow-up studies in NOD mice where metabolic phenotypes, tissue-specific metabolome and transcriptome as well as gut microbiota are characterized in the context of early disease pathogenesis.

## Results

### Longitudinal serum lipidomics in pre-diabetic NOD mice

**Figure 2:**
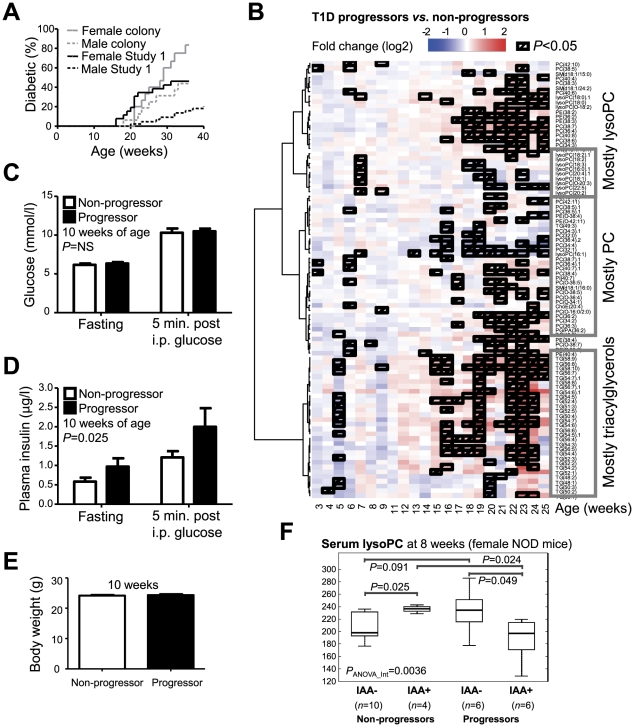
Normoglycemic female NOD mice which later progress to diabetes have elevated glucose stimulated plasma insulin and diminished lipids at an early age. (**A**) Incidence of diabetes in female (*n* = 26) and male (*n* = 44) NOD mice included in the longitudinal lipidomics study. The cumulative incidence of diabetes in this study was lower than the colony incidence of 80% in females and 35% in males. (**B**) Age-dependent progression of lipidomic profiles in females, viewed as ratios of mean lipid concentrations of diabetes progressors (*n* = 12) *vs.* non-progressors (*n* = 14). The hierarchical clustering of lipids was performed across all 733 samples analyzed. PC, phosphatidylcholine; lysoPC, lysophosphatidylcholine. (**C**) Blood glucose levels in 10-week-old female NOD progressors (*n* = 11) and non-progressors (*n* = 14) after 4 hr fast and 5 minutes after intraperitoneal (i.p.) glucose (1 g/kg) administration (2-way ANOVA with glucose administration and diabetes progression as factors, reported *P*-value for diabetes progression; error bars ± SEM). (**D**) Plasma insulin concentrations (mice and statistic same as in panel **C**). (**E**) There were no differences in body weight between the groups (mice and statistic same as in panel **C**). (**F**) Concentration of serum lysophosphatidylcholine (lysoPC; measured as total added concentration of PC(16∶0/0∶0) and PC(18∶0/0∶0)) in 8-week female NOD mice as dependent on diabetes progression and insulin autoantibody (IAA) positivity. Surrogate marker derived from lysoPC level and IAA positivity (Figure S2) was used to stratify mice according to diabetes risk in subsequent studies where mice were sacrificed for tissue-specific studies. Serum lipidomics, insulin, glucose, and body weight measurements were independently repeated in three independent studies (Studies 2–4; see Figures 6 and 7).

Our first objective was to validate whether the NOD mouse was a good model of T1D able to recapitulate the lipidomic-based metabolic phenotypes observed in the longitudinal study of children who later progressed to T1D (Type 1 Diabetes Prediction and Prevention project; DIPP) [2], [8]. Hence we performed a murine study using NOD mice and using a similar protocol as applied in human studies (Study 1). A total of 70 NOD/Bom mice (26 female) were monitored weekly with serum collection from age 3 weeks until either (a) the development of diabetes (progressor group), or (b) followed until 36 weeks of age in females and 40 weeks in males in the absence of a diabetic phenotype (non-progressor group) (Figure 2A). Similarly as in the DIPP study [2], we were primarily interested in early pre-diabetic differences of lipidomic profiles, in mice of the same colony, between diabetes progressors and non-progressors.

Lipidomic analysis using established methodology based on Ultra Performance Liquid Chromatography (UPLC) coupled to mass spectrometry (MS) [14] was performed on a complete sample series from 26 female mice (12 progressors, 14 non-progressors) and 13 male (7 progressors, 6 non-progressors) mice, comprising a total of 1172 samples or 30 samples/mouse on an average (733 samples from female and 439 from male mice), with 154 lipids measured in each sample. When comparing the lipid concentrations of diabetes progressors and non-progressors, the first weeks of life (3–10 weeks) were characterized by an overall lipid-lowering trend among the female progressors, while the period close to the disease onset (15 week and older) was characterized by elevated triglycerides and phospholipids (Figure 2B). No such changes were observed in male mice (Figure S1). The NOD female progressors had similar levels of glycemia (Figure 2C) than the non-progressors but to our surprise the progressors exhibited higher fasting as well as glucose-stimulated plasma insulin levels (2-way ANOVA p = 0.025 for diabetes progression) (Figure 2D) despite the fact that no body weight differences were evident between progressors and non-progressors at 10 weeks of age (Figure 2E). To account for multiple comparisons, false discovery rates among significantly differing lipids were estimated using q-values [15], [16].

Together, these results imply that the mice who later progress to diabetes are characterized by enhanced glucose-stimulated insulin secretion (GSIS) at an early age or that they are inappropriately insulin resistant for their degree of body weight. In fact this increased GSIS associated to early evolutive stages towards T1D is consistent with our earlier findings indicating that the children who later progress to diabetes are characterized by low serum ketoleucine and elevated levels of the more insulinotropic aminoacid leucine prior to seroconversion to insulin autoantibody (IAA) positivity [2], [17].

### Mapping of human and NOD mouse pre-diabetic lipidomic profiles

In order to systematically investigate similarities between early metabolic phenotypes of autoimmune diabetes progressors in mice and men, we proceeded with comparative analysis of longitudinal lipidomic profiles from female NOD mice and DIPP study children [2]. One inherent challenge in the studies of early disease pathophysiology is variable disease penetration. The metabolic profiles may individually change at different paces, and it is not obvious how they should be compared between individuals or species in the context of the disease process. We recently introduced a concept that the maturation of metabolic profiles with age, such as during normal development or early disease pathogenesis, can be described in terms of *metabolic states* derived using the Hidden Markov Model (HMM) methodology [18]. Instead of observing progression of average lipidomic profiles (Figure 2B), our approach allows for each individual's lipidomic profiles to mature at their own pace. Such individual profiles are captured into a set of progressive HMM states (described by mean lipid profiles) using an underlying statistical model.

**Figure 3:**
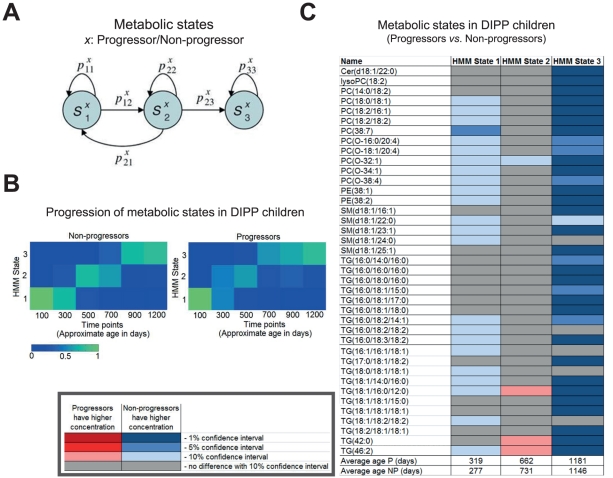
Progression of metabolic states in children who later progress to type 1 diabetes as compared to non-progressors, based on lipidomics data from an earlier study [**2**]. (**A**) Structure of the Hidden Markov Model (HMM) used in this study. The model is made to focus on progressive changes of lipidomic profiles over time [18] by assuming that returning back in states is not possible after State 2. Separate HMM models were developed for progressors and non-progressors. The nodes in the graph represent the hidden states, each of which emits a multivariate profile of metabolite concentrations, while arrows represent possible transitions between the states. (**B**) HMM state progression as a function of age is similar for progressors and non-progressors. Each column shows the probabilities of being in the three states at a certain age, estimated by bootstrap. (**C**) Differences in lipidomic profiles (mean lipid concentrations) between progressors and non-progressors as a function of the progressive metabolic state, colored according to bootstrap-based confidence intervals.

Firstly, we proceeded with the analysis of previously reported longitudinal lipidomic profile data from the DIPP study children [2]. The nested case-control study included 56 T1D progressors and 73 matched non-progressors, comprising a total of 1196 samples or 9.3 samples per child on average between birth and the diagnosis of T1D (in progressors). We applied the HMM methodology to study the longitudinal lipidomic profiles in DIPP children and identified a three-state HMM, developed separately for T1D progressors and non-progressors, to describe the progression of metabolic states at early ages (up to 3 years) (Figure 3A,B). As expected based on the earlier report [2], the first state corresponding to the first year of life was characterized by low triglycerides and specific phospholipids in T1D progressors (Figure 3C). In both progressors and non-progressors the states followed a similar time course (Figure 3B), but the first and third states, corresponding to the first and third years of life, respectively, were qualitatively different between the two groups. On the other hand, there were no such clear differences in the second state, corresponding to the second year of life in average. Such multi-stage progression of lipidomic profiles during the first 3 years of life was not detected when examining them cross-sectionally in different age cohorts.

**Figure 4:**
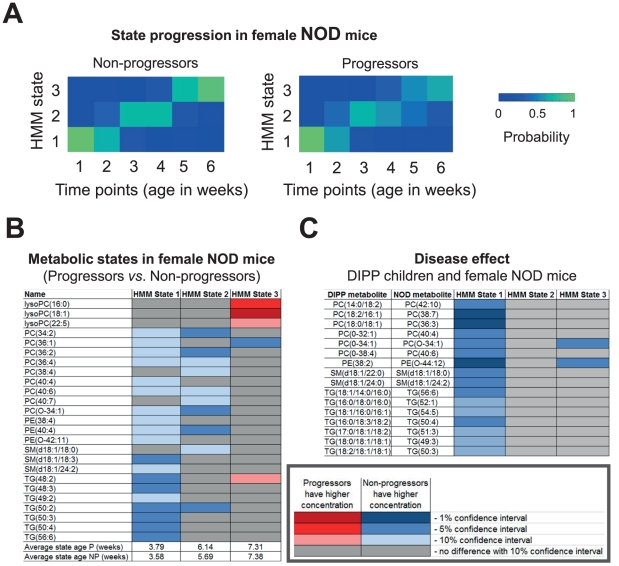
Similarities between lipid changes observed in children who later progress to T1D and the early prediabetic changes present in female NOD mouse progressors. (**A**) HMM state progression as a function of age in female NOD mice is similar for progressors and non-progressors. Each column shows the probabilities of being in the three states at a certain age, estimated by bootstrap. (**B**) Differences in lipidomic profiles (mean lipid concentrations) between progressors and non-progressors as a function of the progressive metabolic state, colored according to bootstrap-based confidence intervals. (**C**) Differences in lipid concentrations in diabetes progressors *vs.* non-progressors that generalize across the species. Mapping shown on the left is inferred from longitudinal lipidomic profiles from DIPP study children, including *n* = 56 progressors and *n* = 73 non-progressors [2] (Figure 3), and NOD female mice (same as in Figure 2).

We then applied the HMM methodology to study the longitudinal lipidomic profiles of female NOD mice and identified a three-state HMM to describe the progression of metabolic states at early ages (3–10 weeks) (Figure 4A,B). The first two states, corresponding to mean ages of approximately 4 weeks and 6 weeks, respectively, were similar to the first state in DIPP children (Figure 3C) and characterized by decreased phospholipids and triglycerides among the progressors (Figure 4B). In the third state, corresponding to approximately 7 weeks of age when a large fraction of the NOD mice already seroconvert to islet autoantibodies [9], the differences observed in the first two states have disappeared. Instead, the levels of proinflammatory lysophosphatidylcholines (lysoPCs) were increased in diabetes progressors (1%–10% confidence interval for progressors having higher concentrations, see Figure 4B).

The similarity of state progression in children (Figure 3) and female NOD mice (Figure 4B) presenting with diabetes suggests that the early disease stages as reflected in the lipidomes share similar metabolic perturbations. However, it is always a challenge to compare species exhibiting differences in systemic lipid metabolism as well as diet-related effects on the lipidomic profiles. Consequently the mapping of molecular lipids between mouse and man may not be trivial and our results should be considered qualitative.

In order to compare progression of mouse and human lipidomic profiles we applied a mapping algorithm [19] that captures their dependencies across the two species. By using this strategy it is possible to compare lipidomic profiles across species, and we therefore sought for the disease effect by a two-way analysis on progressors/non-progressors *vs.* men/mice. By this approach, we uncovered associations of functionally and structurally related lipids between the species (Figure 4C) and confirmed strong association of diminished phospholipids with the development of the disease at an early age (HMM state 1). We can thus conclude that the lipid changes seen in children prior to the first seroconversion to autoantibodies are also characteristic of the early changes in female NOD mice progressors.

### Lysophosphatidylcholine and IAA in early diabetes progression

Seroconversion to islet autoantibody positivity is associated with transiently elevated lysoPC serum levels in children who subsequently progress to T1D [2]. Here we measured the IAA levels in NOD mice at 8 weeks of age and similarly confirmed that the IAA-negative (IAA−) progressor female NOD mice had elevated lysoPC as compared to IAA- non-progressors at a marginal significance level (p = 0.091, see Figure 2F). Intriguingly, IAA positivity had the opposite association with diabetes progression since the IAA-positive (IAA+) mice with high lysoPC were protected from diabetes (Figure 2F). It can be speculated that due to their opposite association with disease progression IAA measurement in combination with lysoPC may help stratify the NOD mice according to their risk of developing diabetes. We derived a surrogate marker by combining autoantibody positivity and lysoPC concentration, which reasonably well discriminated between progressors and non-progressors (χ^2^ = 5.75, *P*_χ2_ = 0.0044; Figure S2), with the NOD mice in the assigned “High-risk” group being at 4.3-fold higher risk (95% lower tolerance bound = 2.6, as calculated from 1000-fold resampling) of developing autoimmune diabetes as compared to the mice in the “Low-risk” group.

### Specific islet and liver pathways associate with T1D risk

**Figure 5:**
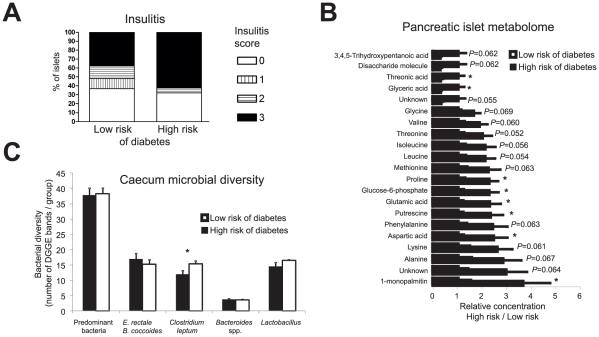
Female NOD mice at high risk of diabetes have more insulitis, elevated levels of insulinotropic amino acis in pancreatic islets, and diminished diversity of *Clostridium leptum* bacteria in caecum. (**A**) Grading of pancreatic islet insulitis in normoglycemic 19-week-old female NOD mice comparing high- and low-risk groups. Insulitis was graded: 0, no visible infiltration, I peri-insulitis, II insulitis with <50% and III insulitis with >50% islet infiltration. 52 islets from four high-risk (11–17 islets/each) and 28 islets from three low-risk mice (7–10 islets/each) were graded. There was a tendency to more severe insulitis in the high-risk group (*P* = 0.07, χ^2^ test). Insulitis scoring was repeated in Study 4 (see Figure 6G). (**B**) Significantly regulated and selected other metabolites (*P*<0.07), out of 125 measured, in islets from female mice at high (HR) *vs.* low risk (LR) of developing diabetes (Study 2). Fourteen mice were 8 weeks old (two IAA+ LR, three IAA− LR, four IAA+ HR, five IAA− HR) and 11 were 19 weeks old (four IAA+ LR, three IAA− LR, one IAA+ HR, three IAA− HR) at time of sacrifice. FDR (Max. *q*-value [15] at *P*<0.05) = 0.12. (**C**) Bacterial diversity of caecum samples from 19-week old female NOD mice, *n* = 4 from HR group and *n* = 7 from LR group, as detected with group specific DGGEs. Bifidobacteria did not amplify from any sample. Islet metabolomics and caecum DGGE analysis were performed once (*i.e.*, only in NOD Study 2).

In an independent experiment normoglycemic female NOD mice from the same colony as in the first experiment were sacrificed at 8 (*n* = 57) or 19 (*n* = 14) weeks of age and blood, liver and pancreas samples were collected (Study 2). We selected sixteen 8-week-old mice (seven were IAA+) and thirteen 19-week-old mice (six were IAA+) for UPLC-MS based serum lipidomics analysis for subsequent risk stratification using the algorithm described above. Mice at high risk of developing diabetes showed a tendency towards more severe insulitis (Figure 5A), therefore providing an independent validation of the surrogate marker. In parallel liver and islet transcriptomics was performed in 19-week-old mice.

When comparing high- and low-risk mice, independent of IAA level, the pathway analysis of islet gene expression data using Gene Set Enrichment Analysis (GSEA) [20] expectedly revealed upregulation of several apoptotic and immunoregulatory pathways in the high-risk group (Table 1 and Table S1). These pathways were associated with the autoimmune status, as they were also upregulated when comparing IAA+ and IAA− mice independent of diabetes risk. In support of our findings from pre-diabetic mice, some of the upregulated gene products of these pathways are in fact known to be implicated in progression to autoimmune diabetes, including CD3 from the CTLA4 pathway [21], pro-inflammatory chemokine CCL5 (or RANTES) from the toll like receptor signalling pathway [22], [23], [24] and the IL-7 pathway [25] (Table S2).

**Table 1:** Pathway analysis in female NOD mouse islets.

	N	NES	FDR *q*	Source
**Upregulated in progressors, associated with IAA positivity**				
HIVNEFPATHWAY	53	2,32	0,000000	BioCarta
HSA04650_NATURAL_KILLER_CELL_MEDIATED_CYTOTOXICITY	94	2,21	0,000157	KEGG
APOPTOSIS_GENMAPP	40	2,18	0,000307	GenMAPP
CELL_CYCLE_KEGG	79	2,16	0,000410	GenMAPP
HSA04660_T_CELL_RECEPTOR_SIGNALING_PATHWAY	90	2,15	0,000413	KEGG
IL7PATHWAY	16	2,04	0,001660	BioCarta
APOPTOSIS	63	2,03	0,001676	GenMAPP
CTLA4PATHWAY	16	2,02	0,001900	BioCarta
HSA03022_BASAL_TRANSCRIPTION_FACTORS	30	2,00	0,002515	KEGG
HSA04662_B_CELL_RECEPTOR_SIGNALING_PATHWAY	59	2,00	0,002531	KEGG
**Upregulated in progressors, not associated with IAA positivity**				
HSA00240_PYRIMIDINE_METABOLISM	82	2,12	0,000763	KEGG
RIBOSOMAL_PROTEINS	71	2,03	0,001743	GenMAPP
HSA04610_COMPLEMENT_AND_COAGULATION_CASCADES	62	1,92	0,004885	KEGG
NKCELLSPATHWAY	18	1,90	0,005527	BioCarta
CARM_ERPATHWAY	24	1,89	0,006187	BioCarta
HSA00100_BIOSYNTHESIS_OF_STEROIDS	21	1,88	0,006819	KEGG
HSA00230_PURINE_METABOLISM	135	1,87	0,008010	KEGG
KREBS_TCA_CYCLE	28	1,85	0,009439	GenMAPP
INTRINSICPATHWAY	22	1,82	0,011212	BioCarta
GLYCOLYSIS_AND_GLUCONEOGENESIS	38	1,81	0,012117	GenMAPP
**Downregulated in IAA positive non-progressors**				
OXIDATIVE_PHOSPHORYLATION	56	−1,76	0,080640	GenMAPP
KREBS_TCA_CYCLE	28	−1,66	0,129344	GenMAPP
MITOCHONDRIAL_FATTY_ACID_BETAOXIDATION	15	−1,61	0,152160	GenMAPP
HSA03010_RIBOSOME	55	−1,57	0,163501	KEGG
HSA00480_GLUTATHIONE_METABOLISM	34	−1,45	0,196112	KEGG
HSA00280_VALINE_LEUCINE_AND_ISOLEUCINE_DEGRADATION	40	−1,46	0,196296	KEGG
**Upregulated in IAA positive non-progressors**				
ST_INTEGRIN_SIGNALING_PATHWAY	78	2,06	0,000774	STKE
HSA05211_RENAL_CELL_CARCINOMA	67	1,99	0,001681	KEGG
INTEGRIN_MEDIATED_CELL_ADHESION_KEGG	90	1,95	0,002570	GenMAPP
IL6PATHWAY	19	1,95	0,002717	BioCarta
SA_PTEN_PATHWAY	16	1,85	0,005938	SigmaAldrich
ST_INTERLEUKIN_4_PATHWAY	23	1,84	0,006384	STKE
SIG_CHEMOTAXIS	44	1,77	0,011176	SignalingAlliance
CELL_GROWTH_AND_OR_MAINTENANCE	58	1,75	0,012905	GO
ECMPATHWAY	20	1,73	0,015894	BioCarta
RAC1PATHWAY	22	1,68	0,021239	BioCarta

Up to 10 most significantly affected pathways are shown at False Discovery Rate (FDR) *q*<0.25 for two different comparisons: (1) High (HR) *vs.* low diabetes risk (LR) and (2) IAA positive LR *vs.* other (Study 2). Full pathway analysis results are shown in Table S1. Transcriptomics was performed in the islets of *n* = 10 19-week old female NOD mice (three IAA+ LR, two IAA− LR, two IAA+ HR, three IAA− HR). N, number of genes in the pathway; NES, normalized enrichment score; Source, gene list source.

Several upregulated pathways in high-risk mice were not associated with the IAA titer. These pathways associated with high risk of developing diabetes were mainly metabolic pathways and included upregulated genes from TCA cycle and glycolysis/gluconeogenesis (Table 1). In order to directly measure the metabolic products of these pathways, we performed metabolomic analysis of islets using two-dimensional gas chromatography coupled to time-of-flight mass spectrometry (GC×GC-TOFMS) [26]. Metabolomics confirmed dysregulation of energy and amino acid metabolism in the islets of high-risk mice (Figure 5B), as several key metabolites of these pathways were found upregulated, including glutamic and aspartic acids, as well as at a marginal significance level all three branched chain amino acids (BCAAs). These elevated amino acids are known insulin secretagogues in β cells [27]. In agreement with this, the insulin signaling pathway was upregulated in the livers of high-risk mice (Table S3). The top ranking gene in this pathway, Glucose-6-phosphatase, catalytic, 2 (G6PC2; fold change high- *vs.* low-risk group +11%, *P* = 0.0034), controls the release of glucose from liver into the bloodstream. However, the animals included in this study, as in the earlier longitudinal study, were normoglycemic and there were no differences in body weight between the two groups. The metabolic changes in β cells and liver can thus explain the observed elevated GSIS in mice at high risk for developing autoimmune diabetes (Figures 2C–E).

### Diminished diversity of gut microbiota associates with diabetes risk

We recently observed that the serum metabolome of germ-free mice is similar to pre-autoimmune metabolomes of children who later progress to T1D [28], thus implying that gut microbiota of T1D progressors may be devoid of important constituents or has an impaired function that predisposes the children to T1D. Given the observed similarities of metabolomes of diabetes progressors in mice and men (Figure 4), we hypothesized that the observed metabolic differences between the high- and low-risk mice may be reflected in differences in their gut microbial composition.

We characterized the microbial composition of caecum samples from high- and low-risk mice from Study 2 using predominant bacterial as well as five different bacterial group-specific (namely *Eubacterium rectale* – *Blautia coccoides* group, *Clostridium leptum* group, *Bacteroides* spp., bifidobacteria, and lactobacilli) denaturing gradient gel electrophoresis (DGGE) methods as previously described [29], [30]. With such an approach to profile microbiota it is possible to detect the phylotypes that constitute over 1% of the specific group in question [29], [31]. Analysis of the composite dataset, which included all the different group-specific DGGE results, showed that the total bacterial composition did not markedly differ between the groups but was slightly more coherent in low-risk mice than in high-risk mice (see the deviation bars in Figure S3). In addition, the high-risk mice had significantly diminished diversity of the *Clostridium leptum* group of the Firmicutes phylum (Figure 5C).

### Markers of insulin resistance in progression to T1D

There is evidence from clinical studies that insulin resistance is a risk factor for progression to T1D [32], [33]. It is also known that the NOD genetic background may predispose the mice to insulin resistance [34]. To test for insulin resistance as a potential explanation for the observed metabolic phenotype of high-risk mice, we performed two independent studies in another NOD colony where (Study 3) 36 female NOD/MrkTac mice were tested for GSIS, glucose and insulin tolerance, and plasma leptin between 8 and 11 weeks of age; and (Study 4) 42 female NOD/MrkTac were sacrificed at 10 weeks of age and tested for insulitis, plasma leptin and adiponectin. As before, serum lipidomics and IAA assays were performed to stratify the mice into high- and low-diabetes-risk groups.

**Figure 6:**
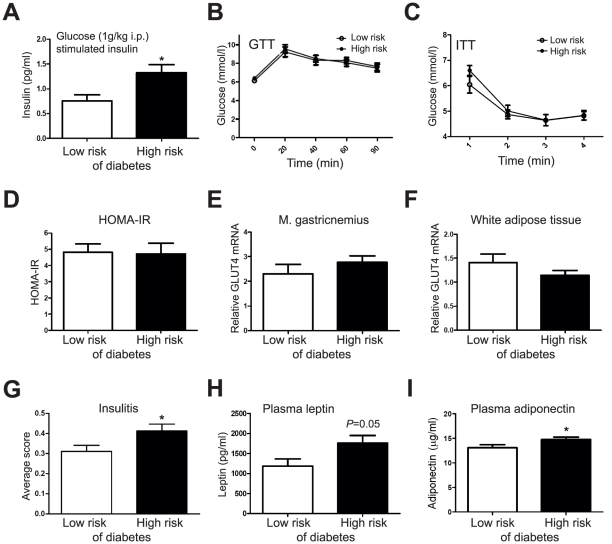
Markers of insulin resistance in 8–11 week old female NOD mice. (**A**) Glucose-stimulated insulin secretion is elevated in the high-risk (HR) group (*n* = 18) as compared to low-risk (LR) group (*n* = 12) (measured in NOD Study 3). In the same mice, no significant differences between the HR and LR group were found in (**B**) glucose tolerance test (GTT) or (**C**) insulin tolerance test (ITT). (**D**) Homeostatic model assessment (HOMA-IR) index in LR (*n* = 13) and HR (*n* = 25) groups (Study 4), and GLUT4 expression in (**E**) muscle and (**F**) white adipose tissue (Study 4). (**G**) The HR mice at 10 weeks of age have slightly more insulitis. Total 678 islets from 8 LR mice (60–123 islets/each) and 633 islets from 8 HR mice (59–102 islets/each) were graded as in Figure 5A. (**H**) Plasma leptin (analyzed twice, in Studies 3 and 4, combined data analyzed; *n* = 24 for LR and *n* = 43 for HR) and (**I**) adiponectin (analyzed in Study 4; *n* = 14 for LR and *n* = 27 for HR) are elevated in 10-week-old HR mice. * indicates p<0.05.

**Figure 7:**
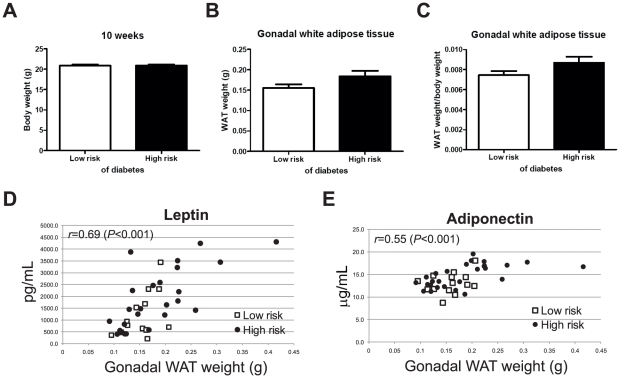
Weight and adiposity in progression to autoimmune diabetes. (**A**) Body weight (Study 4; *n* = 15 for lor-risk and *n* = 27 for high-risk mice). (**B**) Weight and (**C**) weight/body weight of gonadal white adipose tissue (Study 4). Correlations of (**D**) leptin and (**E**) adiponectin with weight of gonadal white adipose tissue. Adiposity was characterized in one independent study (Study 4).

We confirmed the elevated GSIS in high risk mice (Figure 6A) but found no significant difference in glucose responses to intraperitoneal glucose or insulin between the groups (Figures 6B,C), in the Homeostatic model assessment of insulin resistance (HOMA-IR) index or GLUT4 expression in white adipose tissue and muscle (Figure 6D,F). In agreement with the results from older mice (Figure 5A) and in further support of the surrogate marker applied to stratify the mice according to disease risk, the 10-week old female NOD mice at higher risk of developing diabetes have already signs of more insulitis than their low-risk counterparts, although the average degree of insulitis is mild in both groups (p<0.05, see Figure 6G). Surprisingly, the adipose tissue derived hormones leptin (p<0.05, see Figure 6H) and adiponectin (p<0.05, see Figure 6I) were both elevated in plasma of high-risk mice despite no significant differences in weight or adiposity (p>0.05, see Figure 7A–C). However, both adiponectin and leptin correlated with the gonadal adipose tissue mass (Figure 7D,E).

### IAA positivity and protection from autoimmune diabetes

Given that the metabolic profile is normalized in children following seroconversion to autoantibody positivity [2], we proposed earlier that the generation of autoantibodies may be a physiological response to early metabolic disturbances. In the present study (mice from Study 2), we investigated the pathways in the IAA+ low-risk female mice and compared them to all other groups. The IAA+ low-risk mice were characterized by several elevated signaling pathways in the islets, including the IL-4 and IL-6 pathways (Table 1). IL-4 is known to be protective from diabetes in NOD mouse [35]. Conversely IAA+ low-risk mice had reduced expression of pathways mainly related to mitochondrial function and TCA cycle, BCAA catabolism, beta oxidation and oxidative phosphorylation. It is unclear how downregulation of these pathways may protect against T1D. However downregulation of these pathways will lead to a state of reduced production of reactive oxygen species (ROS) [36] which may explain at least in part the conserved β cell functionality. This would offer a potential protective mechanism linking decreased ROS production to the prevention of β cell apoptosis in IAA+ mice which do not progress to diabetes. Our results stress the need for similar studies in terms of protection from diabetes in individuals who seroconverted but did not progress to overt disease.

## Discussion

This study emphasizes the translatability of our previous findings from the large-scale clinical study [2] into the tissue-specific context. Also, our study highlights that specific metabolic disturbances are identifiable early on during the evolutive stages and could potentially be linked to pathogenic mechanisms implicated in the progression to autoimmune diabetes. Although the specific causes, likely to be diverse amongst humans and between the NOD mouse and humans, of these early metabolic disturbances remain to be established, our findings pave the way for studies focused on how the metabolism and the immune system interact in early stages of the disease.

The lipidomic profiles associated with progression to T1D in children [2] were similar to early lipidomic profiles in female but not male NOD mice that later progressed to autoimmune diabetes. It is known that female NOD mice are more likely to progress to autoimmune diabetes [9] although the reasons for this are poorly understood. Notably, in humans the T1D incidence is nearly 2-fold higher in men than women [37]. In the present study, we have not pursued the reasons for the gender-specific metabolic differences in NOD mice and have instead focused on studies in female mice since they displayed the similar metabolic patters as observed in human studies.

Both in man and mouse, the metabolic states as determined by HMM followed the similar progression in disease progressors and non-progressors (Figures 3B and 4A), but were qualitatively different between these two groups (Figures 3C and 4B). However, notably no major qualitative differences were observed in the second state in the human study and in the third state of the mouse study. These two states correspond to the ages when the first diabetes-associated autoantibodies have appeared in many of the human T1D progressors [2] and NOD mice [9], respectively. In the DIPP study we have previously shown that the seroconversion to autoantibody positivity appears to normalize the metabolic profiles, suggesting that the immune system may be involved in the metabolic regulation and *vice versa*. In fact, the metabolic demands of T cells are extraordinary, rivaling that of cancer cells [38], [39]. For example, differentiating T cells consume 10-fold more glutamine than other cells in the body [39], and we in fact found that glutamine is diminished in children within a period of months prior to seroconversion [2]. Concentration changes of circulating metabolites as detected in our studies may thus have a direct effect on T-cell function. We therefore hypothesize that the second metabolic state in human progressors, and similarly the third state in NOD mice, reflect the period following the seroconversion when the metabolic profiles have been restored to normal levels *via* interaction with the immune system.

In the NOD mice, this apparent interaction between the immune system and metabolic status is underlined by the opposite association of the IAA and lysoPC (Figure 2F) at 8 weeks of age, *i.e.*, in the age group corresponding to the third state in the HMM model (Figure 4B). Based on this observation, a surrogate marker was derived combining information on IAA positivity and lysoPC concentration (Figure S2), which was utilized to stratify mice according to risk of developing autoimmune diabetes in subsequent studies. The so-classified high-risk mice had higher degree of insulitis, a histopathological hallmark of progression to diabetes in NOD mice [9], as assessed in two independent studies (Figures 5A and 6G). While the association of the surrogate marker with established characteristics of progression to T1D validated our approach in the present study, it also suggests that prediction of autoimmune diabetes in NOD mice using combined metabolic and immune markers may be feasible. However, further prospective studies are needed in different NOD strains, similar in design as our Study 1, to determine and validate such markers. As already demonstrated in our study, the use of such markers sensitive to disease risk may facilitate investigations of early pathophysiological phenomena at a tissue-specific level prior to any symptoms of the disease.

Our results indicate that early stages of progression to T1D are characterized by acute increased response to high glucose-stimulated insulin secretion. Furthermore, this response is accompanied by increased concentration of insulinotropic amino acids and other markers of energy metabolism in the islets and more specifically of insulin signaling pathways in the liver. Together with human data [2], our study provides compelling evidence that increased GSIS is an event that heralds diabetes progression already in pre-autoimmune stages of the disease pathogenesis. In NOD mice, elevated GSIS at young age may be an initial response associated with early insulitis. Our data suggest that this response might reflect a state of insulin resistance; however our insulin tolerance tests do not support this insulin-resistant component in diabetes progressors. One potential link may be increased circulating insulin concentrations-suppressing leptin [40] and insulin-sensitizing adiponectin [41]. Adiponectin is known to be elevated in patients with T1D, but very limited data exist on its levels during the pre-diabetic period [42]. Leptin, however, is known as an important immune regulator [43]. Leptin is a negative regulator of CD4^+^CD25^+^ regulatory T cells [44] and promoter of Th1 immune responses [45], [46]. In fact administration of leptin accelerates autoimmune diabetes in female NOD mice [47]. Of interest, endoplasmic reticulum stress is known to induce leptin resistance [48]. Together, our findings from the studies of female NOD mice at high- and low-risk of T1D within the same colony suggest that elevated leptin in high-risk mice is a consequence of early metabolic stress, and that leptin may play a role in mobilization of deleterious Th1 immune responses characteristic of T1D [49]. This would offer an explanation for the epidemiological findings that obesity [50] and decreased insulin sensitivity [51] are risk factors of T1D. Given the global rise of obesity and related metabolic complications among children [52], our study thus suggests that improving insulin sensitivity while avoiding harmful immune responses in genetically susceptible individuals may be an important new strategy for early T1D prevention.

Our study also implicates that early metabolic disturbances in progression to autoimmune diabetes associate with diminished diversity of specific bacterial groups such as *C. leptum* group. This is in agreement with a recent pilot study in the DIPP cohort where phylum Firmicutes was found decreased in the four children who later progressed to diabetes [53]. Microbial communities are sensitive to disturbances and may subsequently not return to the their original state [54]. Interestingly, diminished diversity of the anti-inflammatory commensal bacterium *Faecalibacterium prausnitzii* from the *C. leptum* group characterizes also Crohn's disease [55]. Our study thus revealed a candidate microbial group which may be further considered in the context of diabetes prevention.

The fact that the diabetes-associated differences in microbial composition were observed among the mice of the same colony suggests that the observed diminished microbial diversity is rather a consequence than a primary cause of immunological or metabolic responses. The mechanisms by which the gut microbial community is modulated by specific metabolic and immune factors associated with progression to T1D are at present unclear and demand further investigation. However, these findings may still be important by having a role in early disease pathogenesis. In fact, recent study revealed that microbes from *C. leptum* group induce regulatory T cells in colonic mucosa [56]. Diminishment of *C. leptum* diversity along with elevated leptin may therefore be two mechanisms which promote negative regulation of CD4^+^CD25^+^ regulatory T cells, and therefore also promote the autoimmune response [57].

Our study uncovered multiple factors which may contribute in parallel to progression towards autoimmune diabetes. It is unlikely that any of them is a primary cause to initiate the disease process. Instead, as an early mathematical model of T1D describing changes in numbers of β-cells, macrophages, and Th-lymphocytes concluded, the “onset of type 1 diabetes is due to a collective, dynamical instability, rather than being caused by a single etiological factor” [58]. In this context, understanding the spatial and temporal balance of different disease-contributing factors is important [59]. The study design such as ours may help identify the early factors contributing to the disease as well as their mutual dependencies.

Finally, the metabolic phenotypes described here could be relevant as end points for studies investigating T1D pathogenesis and/or responses to interventions. By proceeding from a clinical study *via* metabolomics and modeling to an experimental model using a similar study design, then evolving further to tissue-specific studies, we hereby present a conceptually novel approach to reversed translation (Figure 1) that may be useful in future therapeutic studies in the context of prevention and treatment of T1D as well as of other diseases.

## Materials and Methods

### Ethics statement

All experimental procedures were approved by the Committee for Laboratory Animal Welfare, University of Turku.

### Experimental animals and sample collection

The mice were kept in an animal room maintained at 21±1°C with a fixed 12∶12 hr light-dark cycle. Standard rodent chow (Special Diet Services, Witham, UK) and water were available ad libitum. The colonies of NOD/Bom mice used were bred and maintained in the animal facilities of University of Turku and originated from mice purchased from Taconic Europe (Ry, Denmark). 26 female and 44 male NOD mice (Study 1) underwent weekly blood sampling by venopuncture from the tail vein starting at 3 weeks of age until the mice developed diabetes (blood glucose ≥14.0 mmol/in two consecutive weeks) or until female mice reached 36 weeks and male mice 40 weeks of age. Serum was separated and quickly frozen in −70°C for metabolomic analysis. Blood samples for detection of insulin autoantibodies (IAA) were collected from tail vein at the age of 8 weeks. Plasma samples for insulin were collected between noon and 2 PM after 4 hr fast and two days later 5 minutes after intraperitoneal glucose (1 g/kg) administration at the age of 10 weeks. Another set of euglycemic NOD/Bom female mice (Study 2) was sacrificed with decapitation under CO_2_ anesthesia at the age of 8 weeks (*n* = 57) or 19 weeks (*n* = 14), and blood, liver and pancreas samples were collected.

Two separate batches (*n* = 36 and 42, Studies 3 and 4) of female NOD/MrcTac were delivered from Taconic USA (Hudson, NY, USA) at 5 weeks of age. In Study 3, intraperitoneal glucose tolerance test was performed after 4 hr fast at 8 weeks of age by administering glucose (10% [wt/vol], 1 g/kg body weight) and measuring tail vein blood glucose and serum insulin. Serum samples for lipidomics and IAA were collected from tail vein at 10 weeks of age. Intraperitoneal insulin tolerance test was performed after 1 hr fast at 11 weeks of age by administering human insulin (1.0 IU/kg body weight, Protaphane, Novo Nordisk, Bagsvaerd, Denmark). In Study 4, mice were sacrificed at 10 weeks of age after 4 hr fast by cardiac puncture under anesthesia. Gonadal white adipose tissue (WAT) depot was carefully dissected and weighted, and was used as a marker of adiposity. Serum samples for IAA, lipidomics and adipokine panel assays, gonadal WAT, gastrocnemius muscle and pancreas samples were collected, and stored at −70°C until analyses. HOMA-IR, an estimate of insulin resistance, was calculated as fasting insulin (µIU/ml)×fasting glucose (mmol/l)/22.5. Statistical significances were analyzed with Student's t-test or two-way ANOVA using GraphPad Prism 4.

### Plasma glucose, insulin, leptin and adiponectin

Blood glucose was measured with Precision Xtra™ Glucose Monitoring Device (Abbott Diabetes Care, IL). Plasma insulin was analyzed with Mouse Ultrasensitive ELISA kit (Mercodia, Uppsala, Sweden) or together with leptin with Milliplex Mouse Adipokine Panel (Millipore, Billerica, MA, USA). Plasma adiponectin was measured with Mouse Adiponectin ELISA kit from Millipore.

### Islet isolation

Pancreatic islets were isolated using Ficoll 400 (Sigma-Aldrich, St Louis, MI, USA) gradient method [60]. In brief, the pancreata were incubated with Collagenase P (0.5 mg/ml, Roche Diagnostics, Mannheim, Germany) in HBSS containing 10 mM HEPES, 1 mM MgCl2, 5 mM Glucose, pH 7.4 for 17 min. After two rounds of washing, the pellet was resuspended in Ficoll 25%, and the densities 23%, 20% and 11% were layered on top. After centrifugation, the islet layer between densities 23% and 20% was collected and washed twice before snap freezing the pellet for metabolomic analysis or homogenization in lysis buffer for RNA extraction. Samples were stored in −70°C until analyses.

### Histopathology of diabetes

Pancreata from euglycemic NOD mice were cryosectioned. 5 µm sections with >20 µm intervals were stained with hematoxylin & eosin and graded for insulitis as follows: 0, no visible infiltration, I peri-insulitis, II insulitis with <50% and III insulitis with >50% islet infiltration. Total 678 islets from eight female 10-week-old low-risk mice (60–123 islets/each) and 633 islets from eight high-risk mice (59–102 islets/each), and 52 islets from four female 19-week-old low-risk mice (11–17 islets/each) and 28 islets from three high-risk mice (7–10 islets/each) were graded. Statistical significance was analyzed with Student's t-test or Chi Square test using GraphPad Prism 4.

### IAA assay

Murine IAA were measured by a radiobinding microassay (RIA) with minor modifications to that previously described for human IAA [61]. Mouse sera (2.5 µl) and serial dilutions of standard samples (5 µl) of a serum pool obtained from persons with a high IAA titer were incubated for 72 h with 15,000 cpm mono^125^I-(TyrA14)-insulin (Amersham, GE Healthcare, Buckinghamshire, UK) in the presence or absence of an excess of unlabeled human recombinant insulin (Roche Diagnostics, Mannheim, Germany). Antibody complexes were precipitated by adding 50 µl TBT buffer (50 mM Tris, pH 8,0, 0,1% Tween 20) containing 8 µl Protein A and 4 µl Protein G Sepharose (Amersham). After repeated washings the bound radioactivity was measured with a liquid scintillation detector (1450 Microbeta Trilux, Perkin Elmer Life Sciences Wallac, Turku, Finland). The specific binding was calculated by subtracting the non-specific binding (excess unlabeled insulin) from total binding and expressed in relative units (RU) based on standard curves run on each plate. The cut-off value for mouse IAA positivity was set at the mean+3SDS in 29 BALB-mice, *i.e.* 0.90 relative units (RU).

### Lipidomic analysis

Serum samples (10 µl) in Eppendorf tubes were spiked with a standard mixture containing 10 lipid compounds at a concentration level of 0.2 µg/sample, and mixed with 10 µl of 0.9% sodium chloride and 100 µl of chloroform∶methanol (2∶1). After 2 min vortexing and 1 hr standing the samples were centrifuged at 10000 rpm for 3 min and 60 µl of the lower organic phase was taken to a vial insert and spiked with 20 µl of three labelled lipid standards at a concentration level of 0.2 µg/sample.

The lipidomics runs were performed on a Waters Q-Tof Premier mass spectrometer combined with an Acquity Ultra Performance LC™ (UPLC; Milford MA). The solvent system consisted of 1) water with 1% 1 M NH_4_Ac and 0.1% HCOOH and 2) LC/MS grade acetonitrile/isopropanol (5∶2) with 1% 1 M NH_4_Ac, 0.1% HCOOH. The gradient run from 65% A/35% B to 100% B took 6 min and the total run time including a 5 min re-equilibration step was 18 min. The column (at 50°C) was an Acquity UPLC™ BEH C18 (1×50 mm, 1.7 µm particles) and the flow rate was 0.200 ml/min. The lipids were profiled using ESI+ mode and the data collected at a mass range of m/z 300–1200. The data was processed by using MZmine software (version 0.60) [62], [63] and the lipid identification was based on an internal spectral library [64]. Data was normalized using the appropriate internal standards as previously described [14], [65].

### Metabolomic analysis by GC×GC-TOFMS

Depending on the protein concentrations of PBS buffered cell solutions, 20–40 µl samples were taken for islet metabolomic analysis. 10 µL of an internal standard labeled palmitic acid-16,16,16-d_3_ (250 mg/l) and 400 µl of methanol solvent were added to the sample. After vortexing for 2 min and incubating for 30 min at room temperature, the supernatant was separated by centrifugation at 10,000 rpm for 5 min. The sample was dried under constant flow of nitrogen gas and derivatized with 25 µl of MOX (1 h, 45°C) and MSTFA (1 hr, 45°C). 5 µl of retention index standard mixture with five alkanes (125 ppm) was added to the metabolite mixture.

Islet samples were analyzed by two-dimensional gas chromatography coupled to time of flight mass spectrometry (GC×GC-TOFMS). The instrument used was a Leco Pegasus 4D (Leco Inc., St. Joseph, MI), equipped with an Agilent GC 6890N from Agilent Technologies (Santa Clara, CA) and a CombiPAL autosampler from CTC Analytics AG (Zwingen, Switzerland). The modulator, secondary oven and time-of-flight mass spectrometer were from Leco Inc. The GC was operated in split mode with a 1∶20 ratio. Helium with a constant pressure of 39.6 psig was used as carrier gas. The first dimension GC column was a non-polar RTX-5 column, 10 m×0.18 mm×0.20 µm (Restek Corp., Bellefonte, PA), coupled to a polar BPX-50 column, 1.50 m×0.10 mm×0.10 µm (SGE Analytical Science, Ringwood, Australia). The temperature program was as follows: initial temperature 50°C, 1 min→295°C, 7°C/min, 3 min. The secondary oven was set to 20°C above the oven temperature. Inlet and transfer line temperatures were set to 260°C. The second dimension separation time was set to 5 s. The mass range used was 45–700 amu and the data collection speed was 100 spectra/second. Raw data were processed using Leco ChromaTOF software, followed by alignment using Guineu software (version 0.7) [66]. The metabolites were identified by using an in-house reference compound library together with The Palisade Complete Mass Spectral Library, 600K Edition (Palisade Mass Spectrometry, Ithaca, NY).

### Gene expression and pathway analysis

RNA extraction from islets was carried out with Rneasy minikit (QIAGEN GmbH, Hilden, Germany) and from liver, skeletal muscle (m. gastrocnemius) and gonadal white adipose tissue with Trizol reagent (Invitrogen, Carlsbad, CA) followed by RNase-free DNase I treatment (QIAGEN GmbH) and purification with Rneasy minikit. Pancreatic islets and liver for microarray analysis were collected from 19-week-old euglycemic female NOD/Bom mice. Skeletal muscle and adipose tissue for GLUT4 mRNA expression were collected from 10-week-old female NOD/MrkTac mice.

GLUT4 mRNA expression in skeletal muscle and gonadal white adipose tissue was measured by quantitative real-time PCR. CDNA synthesis was performed with High Capacity RNA-to-cDNA Kit according to manufacturer's protocol. Real-time PCR was performed with 7300 Real Time PCR system, pre-designed TaqMan® Gene Expression Assay for GLUT4 and TaqMan® Endogenous Control Assay for β-actin. The 20 µl PCR reactions contained 8 µl cDNA, 8 µl TaqMan® Gene Expression Master Mix, 1 µl GLUT4 TaqMan Gene Expression Assay, 1 µl b-actin TaqMan Endogenous control Assay and 2 µl depc water. Cycling parameters for real-time RT-PCR were as follows: 50°C for 2 min, 95°C for 10 min followed by 40 cycles of 95°C for 15 seconds and 60°C for one minute. GLUT4 mRNA levels were expressed relative to β-actin, which was used as a housekeeping gene. Relative gene expression was calculated using the comparative CT method and RQ = 2^−ΔΔCT^ formula. All reagents were from Applied Biosystems (Foster City, CA, USA).

RNA amplification was performed from 300 ng total RNA with Ambion's (Austin, TX) Illumina RNA TotalPrep Amplification kit (cat no AMIL1791). IVT reaction overnight (14 hr), during it cRNA was biotinylated. Both before and after the amplifications the RNA/cRNA concentrations where checked with Nanodrop ND-1000 (Wilmington, DE) and RNA/cRNA quality was controlled by BioRad's Experion electrophoresis station (Hercules, CA).

The samples were hybridized in the Finnish DNA Microarray Centre, at the Turku Centre for Biotechnology. 1.50 µg each sample was hybridized to Illumina's MouseWG-6 Expression BeadChips, version 2 (BD-201-0602) at 58°C overnight (18 hr) according to Illumina® Whole-Genome Gene Expression Direct Hybridization protocol, revision A. Hybridization was detected with 1 µg/ml Cyanine3-streptavidine, GE Healthcare Limited (Chalfont, UK) (cat no PA43001). Chips were scanned with Illumina BeadArray Reader, BeadScan software version 3.5. The numerical results were extracted with Illumina's GenomeStudio software *v.* 1.0 without any normalization.

Bead Summary data, exported from Illumina's GenomeStudio software, was preprocessed using *beadarray* package [67] of R/Bioconductor [68] as follows. Data was transformed to logarithm (base 2), and normalized using quantile method [69], which equalizes the distribution of probe intensities across a set of microarrays.

### Pathway analysis and clustering

Gene Set Enrichment Analysis (GSEA) [20], a commonly used pathway analysis technique for microarray gene expression data analysis, uses a Kolmogorov-Smirnov like statistic to test whether selected gene sets are enriched among the most up or down regulated genes. Multiple hypothesis testing was addressed by computing the false discovery rate q-values based on random permutation of membership of genes across gene sets as implemented in the GSEA software [20]. Linear Models for Microarray Data (LIMMA) approach [70] identifies differentially expressed genes by fitting a linear model to the expression data of each gene, and computing moderated t-statistic using posterior residual standard deviations to account for the gene-specific variability of expression values. Here, we used the R/Bioconductor package [68] and LIMMA [70] for testing differential expression of genes. We then performed pre-ranked GSEA analysis using the moderated t-statistic for ranking the gene list, to test for enrichment of gene sets from a variety of pathway databases such as Gene Ontology (GO) [71], GenMAPP [72], BioCarta (http://www.biocarta.com), Signal Transduction Knowledge Environment (STKE) (http://stke.sciencemag.org/), and KEGG [73] curated in Molecular Signatures Database (MSigDB) [20].

Leading edge genes of an enriched pathway are the genes that account for the enrichment signal [20]. For selected pathways that are found statistically significant by GSEA, the pathway profiles are calculated as average expression of all leading edge genes. This matrix of pathway profiles of selected pathways was then augmented with selected metabolite profiles. Then the numerical values in this matrix were normalized with the autoantibody-negative low-risk group (IAA− & LR), *i.e.*, each numerical value of a variable is divided by the average values from IAA− & LR samples, and transformed to logarithmic (base 2) scale. Then the variables were scaled for unit variance. Finally, hierarchical clustering was applied using Euclidean metric and complete linkage method [74] for computing inter-cluster distances. An R package called *gplots* (http://www.r-project.org/) was used for the clustering and displaying the numerical values as a heat map.

### Microbiological analysis

DNA was extracted from 200 mg of fecal sample from caecum using FastDNA Spin Kit for Soil (QBIOgene, Carlsbad, CA,) with modifications to the manufacturer's instructions [29]. PCR-DGGEs of predominant bacterial PCR-DGGE and five different group specific PCR-DGGEs (bifidobacteria, *Lactobacillus*-group, *Eubacterium rectale* – *Blautia coccoides* clostridial group (Erec-group), *Clostridium leptum* clostridial group (Clept group), and genus *Bacteroides*) were performed as described previously [30].

The comparison of the profiles and the quantification of the amplicons were performed using BioNumerics software version 5.1 (Applied Maths NV, Sint-Martens-Latem, Belgium). The statistical analysis of amplicon numbers was performed with the Student's t-test with unequal variances. Clustering was performed with Pearson correlation from each bacterial group separately besides using composite datasets (included predominant bacterial DGGE and five group specific DGGEs) in which amplicons with the total surface area of at least 1% were included in the similarity analysis. Principal component analysis was performed with the BioNumerics software.

### Statistical analysis of lipidomics data

R statistical software (http://www.r-project.org/) was used for data analyses and visualization. The concentrations were compared using the Wilcoxon rank-sum test, with p-values <0.05 considered statistically significant. Due to the large number of tests, one test for each of the 154 lipids, for the differences in mean concentrations between the progressor and non-progressor groups some p-values may be small due to chance. In order to quantify the number of such false significant findings we estimated the false discovery rates using q-values [15], [16]. A q-value is associated for each lipid with the interpretation that among those lipids that have p-value less than or equal to the p-value of the lipid a fraction q are falsely stated significant. To account for multiple comparisons, false discovery rates among significantly differing lipids were estimated using *q*-values [15], [16]. False discovery rates were computed using the R package q-value. The fold difference was calculated by dividing the median concentration in high-risk group by the median concentration in low-diabetes-risk group and taking the base-2-log of the resulting value. This makes interpretation easy as values greater/smaller than zero correspond to up/down-regulated lipids in the high-risk group. In clustering we applied a customized correlation based distance metric
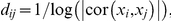
Where 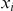 and 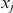 denote the concentrations of lipids 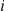 an 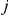 in the sample set. Ward's method was then applied in hierarchical clustering using this distance measure [75].

### Hidden Markov Model of metabolic state progression

Metabolic state development in diabetes progressors and non-progressors was modeled by separate Hidden Markov Models [18], making it possible to align individuals based on metabolic states instead of age, and to compare the metabolic states in progressors and non-progressors. The modeling assumptions under which the models are fitted to data are that individuals share a similar developmental progression but the timing of the states may vary, and that metabolite profiles in each state may be different for progressors and non-progressors. Model fitting was done by the standard Baum-Welch algorithm using the MATLAB toolbox by Kevin Murphy (http://www.cs.ubc.ca/~murphyk/Software/HMM/hmm.html). The model structure was validated by the bootstrap in the same way as in our earlier studies [18], and confidence intervals were estimated with non-parametric bootstrap (5000 samples).

### Mapping of human and mouse metabolites

Let 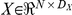 and 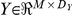 be two data matrices with 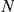 and 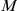 samples, 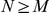, and dimensions 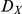 and 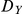, respectively. The task is to find a permutation 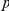 of samples in 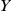 such that each sample 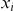 in 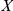 is matched with 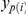 in 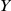. We assume a one-to-one matching of samples between the two data matrices. Since the data matrices do not lie in the same data space, it is not possible to use distance as the matching criterion. We have recently introduced a methodology based on statistical dependencies between the data sets to solve this problem [19]. The idea is to compute from the data features or statistical descriptors that maximize statistical dependencies, and do the matching based on the descriptors. In practice, we project the data onto a lower-dimensional subspace such that the statistical dependencies between the datasets are maximized, and find a matching of samples in this comparable subspace.

### Bootstrap-based two-way analysis

In order to find disease effects shared by NOD mice and humans in the DIPP study, we first paired metabolites of the two organisms, then estimated the metabolic states of progressor and non-progressor men and mice by HMMs, and finally did a bootstrap-based two-way analysis on progressors/non-progressors *vs.* men/mice to identify disease and organism effects and their interactions. The data-driven pairing or the metabolites and the four HMMs were computed as described above. The two-way analysis of disease effect was done by first removing the organism effect, represented with a single mean parameter estimated by least squares, and then computing bootstrap confidence intervals for the disease effect of pooled men and mice. Organism and cross effects were estimated analogously.

## Supporting Information

Figure S1 **Lipidomic profiles of male NOD progressors do not differ from non-progressors.** Age- dependent progression of lipidomic profiles in NOD male mice, viewed as ratios of mean lipid concentrations of diabetes progressors (*n* = 7) *vs.* the non-progressors (*n* = 6). The hierarchical clustering of lipids was performed across all 439 samples analyzed from male NOD mice.
(PDF)

Figure S2 **Surrogate marker for stratifying female NOD mice into two groups with high- and low-risk of developing autoimmune diabetes.** The marker is derived from lysophosphatidylcholine and IAA measurement from 8 week old female mice (same mice as shown in Figure 2F), including *n* = 12 diabetes progressors *vs. n* = 14 non-progressors. The biomarker development assay was applied once (Study 1), and applied in three subsequent independent studies (Studies 2–4). The following algorithm was applied: 1. Calculate lysoPC concentration (µmol/l) as a sum of concentrations of PC(16∶0/0∶0) and PC(18∶0/0∶0). 2. Scale the lysoPC concentration to zero mean and unit variance→lysoPC_S_. 3. Marker calculation. a. If IAA−, then **Marker** = lysoPC_S_. b. If IAA+, then **Marker** = −lysoPC_S_. 4. Estimation of progressors (**P**) and non-progressors (**NP**). a. If **Marker** ≥−0.1 then **P**, else **NP**.
(PDF)

Figure S3 **Microbial composition of caecum in 19-week-old female NOD mice, comparing high- and low-risk groups.** Principal Components Analysis plot of the composite DGGE dataset, which was calculated based on DGGE-profiles of predominant bacteria, *E. rectale* – *B. coccoides* group, *C. leptum* group, *Bacteroides* spp. and *Lactobacillus*-group, bifidobacteria didn't amplify. (star = high diabetes risk, dot = low diabetes risk). *n* = 4 from HR group and *n* = 7 from LR group. DGGE analysis were performed once (*i.e.*, only in NOD Study 2; same mice as Figure 5C).
(PDF)

Table S1 **Pathway analysis of islet transcriptomics data.** Gene Set Enrichment Analysis [20] results at FDR *q*<0.25 for three different comparisons: (1) Progressors (P) *vs.* Non-progressors (NP); (2) IAA+ *vs.* IAA−; (3) IAA+ non-progressors *vs.* other. Transcriptomics was performed on *n* = 10 19-week old female NOD mice (three IAA+ NP, two IAA− NP, two IAA+ P, three IAA− P). N, number of genes in the pathway; NES, normalized enrichment score; FDR *q*, False Discovery Rate *q*-value [15]; Source, gene list source.
(PDF)

Table S2 **Leading edge genes in selected islet pathways.** Leading edge genes for selected upregulated pathways (diabetes progressors *vs.* non-progressors in female NOD mice; Table 1 and Table S1) derived from Gene Set Enrichment Analysis [20] of islet gene expression data. NES, normalized enrichment score; FDR *q*, False Discovery Rate *q*-value [15]; Probe ID, Illumina Mouse WG-6 Expression BeadChips probe ID; Fold, fold change progressors *vs.* non-progressors; *P*-value (*t* statistic); Gene, common gene name.
(PDF)

Table S3 **Pathway analysis of liver transcriptomics data.** Gene Set Enrichment Analysis [20] results at FDR *q*<0.25 for three different comparisons: (1) High diabetes risk (HR) *vs.* low risk (LR); (2) IAA+ *vs.* IAA−; (3) IAA+ LR *vs.* other. Transcriptomics was performed on *n* = 12 19-week old female NOD mice (four IAA+ LR, three IAA− LR, two IAA+ HR, three IAA− HR). N, number of genes in the pathway; NES, normalized enrichment score; FDR *q*, False Discovery Rate *q*-value [15]; Source, gene list source.
(PDF)
