# Supplementary figures and images for: Metabolic Regulation in Progression to Autoimmune Diabetes
Source: PLoS Comput Biol. 2011 Oct 27;7(10):e1002257. doi: 10.1371/journal.pcbi.1002257 (PMC3203065; doi:10.1371/journal.pcbi.1002257)

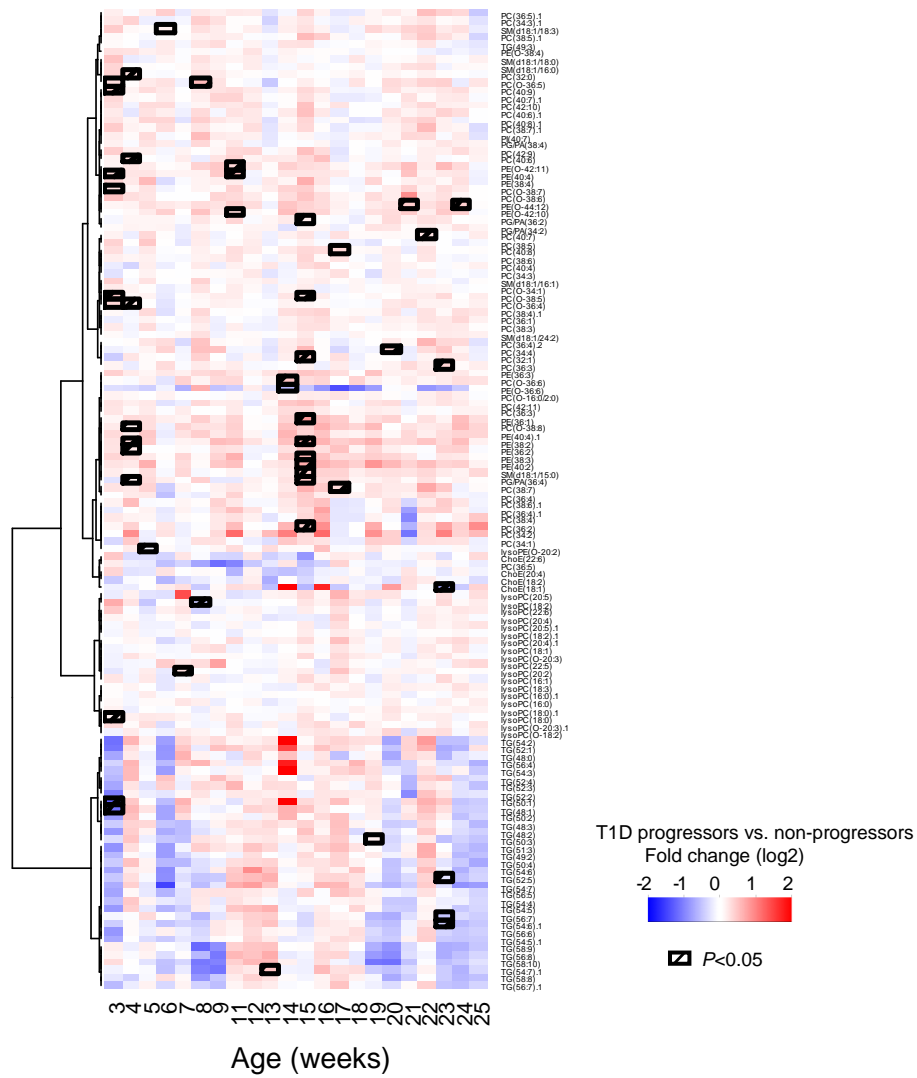

Supplement: Figure S1 — Lipidomic profiles of male NOD progressors do not differ from non-progressors. Age- dependent progression of lipidomic profiles in NOD male mice, viewed as ratios of mean lipid concentrations of diabetes progressors (n = 7) vs. the non-progressors (n = 6). The hierarchical clustering of lipids was performed across all 439 samples analyzed from male NOD mice. (PDF) [file pcbi.1002257.s001.pdf]

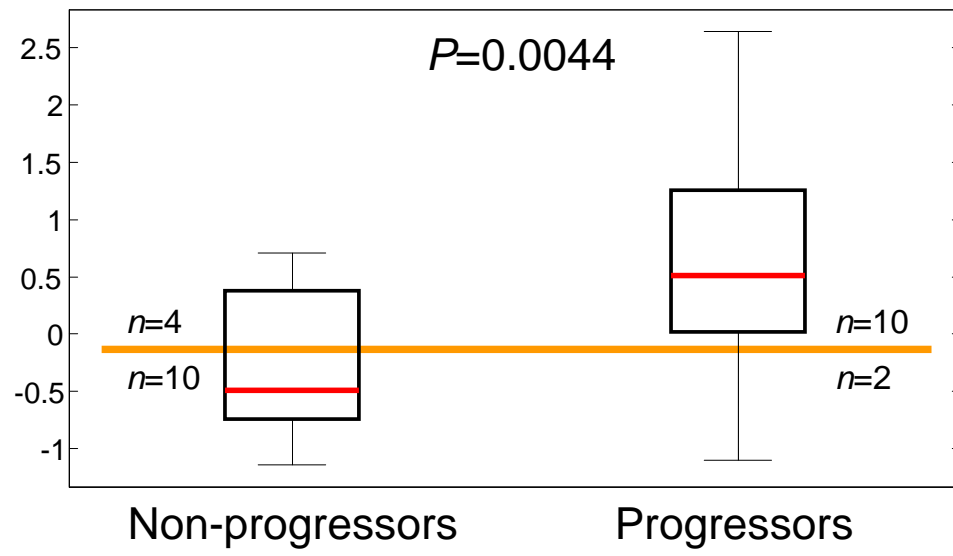

Supplement: Figure S2 — Surrogate marker for stratifying female NOD mice into two groups with high- and low-risk of developing autoimmune diabetes. The marker is derived from lysophosphatidylcholine and IAA measurement from 8 week old female mice (same mice as shown in Figure 2F), including n = 12 diabetes progressors vs. n = 14 non-progressors. The biomarker development assay was applied once (Study 1), and applied in three subsequent independent studies (Studies 2–4). The following algorithm was applied: 1. Calculate lysoPC concentration (µmol/l) as a sum of concentrations of PC(16∶0/0∶0) and PC(18∶0/0∶0). 2. Scale the lysoPC concentration to zero mean and unit variance→lysoPCS. 3. Marker calculation. a. If IAA−, then Marker = lysoPCS. b. If IAA+, then Marker = −lysoPCS. 4. Estimation of progressors (P) and non-progressors (NP). a. If Marker ≥−0.1 then P, else NP. (PDF) [file pcbi.1002257.s002.pdf]

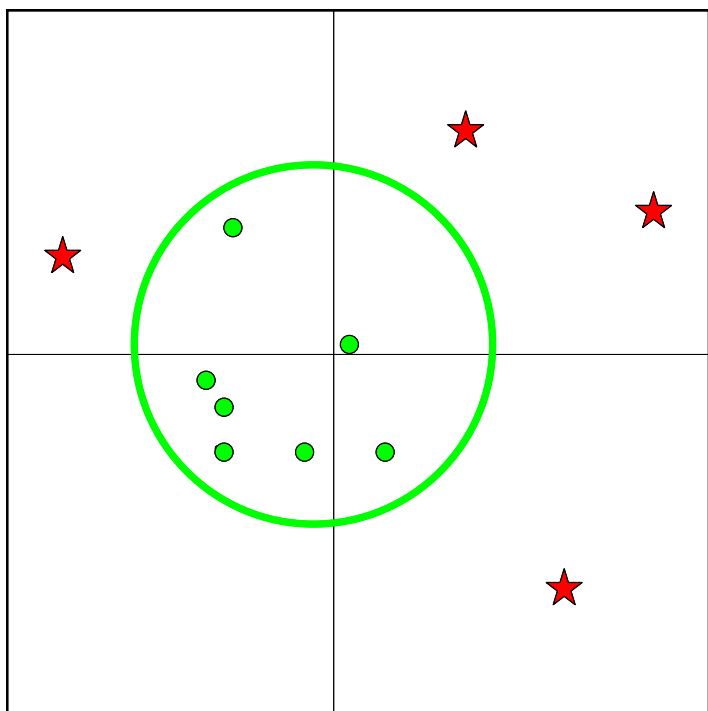

Supplement: Figure S3 — Microbial composition of caecum in 19-week-old female NOD mice, comparing high- and low-risk groups. Principal Components Analysis plot of the composite DGGE dataset, which was calculated based on DGGE-profiles of predominant bacteria, E. rectale – B. coccoides group, C. leptum group, Bacteroides spp. and Lactobacillus-group, bifidobacteria didn't amplify. (star = high diabetes risk, dot = low diabetes risk). n = 4 from HR group and n = 7 from LR group. DGGE analysis were performed once (i.e., only in NOD Study 2; same mice as Figure 5C). (PDF) [file pcbi.1002257.s003.pdf]
