# Supplementary material for: Metabolic Regulation in Progression to Autoimmune Diabetes
Source: PLoS Comput Biol. 2011 Oct 27;7(10):e1002257. doi: 10.1371/journal.pcbi.1002257 (PMC3203065; doi:10.1371/journal.pcbi.1002257)
Supplement: Table S1 — Pathway analysis of islet transcriptomics data. Gene Set Enrichment Analysis [20] results at FDR q<0.25 for three different comparisons: (1) Progressors (P) vs. Non-progressors (NP); (2) IAA+ vs. IAA−; (3) IAA+ non-progressors vs. other. Transcriptomics was performed on n = 10 19-week old female NOD mice (three IAA+ NP, two IAA− NP, two IAA+ P, three IAA− P). N, number of genes in the pathway; NES, normalized enrichment score; FDR q, False Discovery Rate q-value [15]; Source, gene list source. (PDF) [file pcbi.1002257.s004.pdf]

Progressors *vs*. non-progressors				
**Pathway**	**N**	**NES**	**FDR *q***	**Source**
HIVNEFPATHWAY	53	2.32	0.000000	BioCarta
HSA04650_NATURAL_KILLER_CELL_MEDIATED_CYTOTOXICITY	94	2.21	0.000157	KEGG
APOPTOSIS_GENMAPP	40	2.18	0.000307	GenMAPP
CELL_CYCLE_KEGG	79	2.16	0.000410	GenMAPP
HSA04660_T_CELL_RECEPTOR_SIGNALING_PATHWAY	90	2.15	0.000413	KEGG
CELL_CYCLE_CHECKPOINT	22	2.16	0.000430	GO
DNA_REPLICATION_REACTOME	40	2.14	0.000576	GenMAPP
MRNA_PROCESSING_REACTOME	102	2.12	0.000739	GenMAPP
HSA00240_PYRIMIDINE_METABOLISM	82	2.12	0.000763	KEGG
CELL_CYCLE	71	2.07	0.001184	GO
APOPTOSIS_KEGG	46	2.06	0.001257	GenMAPP
IL7PATHWAY	16	2.04	0.001660	BioCarta
APOPTOSIS	63	2.03	0.001676	GenMAPP
RIBOSOMAL_PROTEINS	71	2.03	0.001743	GenMAPP
CTLA4PATHWAY	16	2.02	0.001900	BioCarta
HSA03022_BASAL_TRANSCRIPTION_FACTORS	30	2.00	0.002515	KEGG
HSA04662_B_CELL_RECEPTOR_SIGNALING_PATHWAY	59	2.00	0.002531	KEGG
ST_T_CELL_SIGNAL_TRANSDUCTION	44	1.98	0.003032	STKE
HSA03010_RIBOSOME	55	1.97	0.003345	KEGG
CASPASEPATHWAY	21	1.95	0.004138	BioCarta
G1_TO_S_CELL_CYCLE_REACTOME	65	1.95	0.004205	GenMAPP
SIG_PIP3_SIGNALING_IN_B_LYMPHOCYTES	33	1.92	0.004876	SignalingAlliance
P53PATHWAY	15	1.92	0.004879	BioCarta
HSA04610_COMPLEMENT_AND_COAGULATION_CASCADES	62	1.92	0.004885	KEGG
HSA04210_APOPTOSIS	75	1.92	0.004911	KEGG
BLOOD_CLOTTING_CASCADE	19	1.92	0.004939	GenMAPP
HSP27PATHWAY	15	1.92	0.004940	BioCarta
HSA04115_P53_SIGNALING_PATHWAY	62	1.92	0.004956	KEGG
DEATHPATHWAY	30	1.92	0.004957	BioCarta
HSA01510_NEURODEGENERATIVE_DISEASES	36	1.92	0.004966	KEGG
TNFR2PATHWAY	18	1.92	0.004992	BioCarta
PYRIMIDINE_METABOLISM	54	1.91	0.005078	GenMAPP
STATIN_PATHWAY_PHARMGKB	17	1.91	0.005088	GenMAPP
HSA04612_ANTIGEN_PROCESSING_AND_PRESENTATION	42	1.93	0.005188	KEGG
NKCELLSPATHWAY	18	1.90	0.005527	BioCarta
FASPATHWAY	26	1.90	0.005691	BioCarta
HSA04110_CELL_CYCLE	106	1.90	0.005738	KEGG
CARM_ERPATHWAY	24	1.89	0.006187	BioCarta
AMIPATHWAY	18	1.89	0.006671	BioCarta
HSA00100_BIOSYNTHESIS_OF_STEROIDS	21	1.88	0.006819	KEGG
MITOCHONDRIAPATHWAY	19	1.88	0.007053	BioCarta
HSA00230_PURINE_METABOLISM	135	1.87	0.008010	KEGG
KREBS_TCA_CYCLE	28	1.85	0.009439	GenMAPP
CSKPATHWAY	18	1.84	0.010104	BioCarta
CERAMIDEPATHWAY	21	1.84	0.010247	BioCarta

INTRINSICPATHWAY	22	1.82	0.011212	BioCarta
HSA00530_AMINOSUGARS_METABOLISM	27	1.82	0.011627	KEGG
GLYCOLYSIS_AND_GLUCONEOGENESIS	38	1.81	0.012117	GenMAPP
HSA05221_ACUTE_MYELOID_LEUKEMIA	50	1.80	0.012917	KEGG
RELAPATHWAY	16	1.79	0.013356	BioCarta
RNA_TRANSCRIPTION_REACTOME	34	1.79	0.013458	GenMAPP
ATRBRCAPATHWAY	18	1.78	0.015101	BioCarta
HSA05222_SMALL_CELL_LUNG_CANCER	83	1.78	0.015184	KEGG
HSA04630_JAK_STAT_SIGNALING_PATHWAY	138	1.76	0.017908	KEGG
TIDPATHWAY	16	1.76	0.018501	BioCarta
SMOOTH_MUSCLE_CONTRACTION	137	1.76	0.018531	GenMAPP
HSA05216_THYROID_CANCER	26	1.76	0.018586	KEGG
G2PATHWAY	21	1.76	0.018671	BioCarta
TNFR1PATHWAY	27	1.75	0.019374	BioCarta
SIG_BCR_SIGNALING_PATHWAY	46	1.74	0.020829	SignalingAlliance
NO2IL12PATHWAY	15	1.74	0.021384	BioCarta
ST_FAS_SIGNALING_PATHWAY	58	1.72	0.023729	STKE
SIG_CHEMOTAXIS	44	1.72	0.024324	SignalingAlliance
HSA04664_FC_EPSILON_RI_SIGNALING_PATHWAY	73	1.72	0.024417	KEGG
SIG_REGULATION_OF_THE_ACTIN_CYTOSKELETON_BY_RHO_GTPASES	34	1.71	0.026058	SignalingAlliance
CALCINEURIN_NF_AT_SIGNALING	86	1.70	0.026530	GEArray
ST_B_CELL_ANTIGEN_RECEPTOR	37	1.70	0.026569	STKE
HSA05215_PROSTATE_CANCER	82	1.69	0.028725	KEGG
BADPATHWAY	20	1.69	0.028837	BioCarta
HSA04640_HEMATOPOIETIC_CELL_LINEAGE	65	1.69	0.030282	KEGG
RHOPATHWAY	29	1.68	0.031851	BioCarta
HSA04720_LONG_TERM_POTENTIATION	62	1.67	0.033378	KEGG
CARBON_FIXATION	18	1.66	0.035371	GenMAPP
MITOCHONDRIAL_FATTY_ACID_BETAOXIDATION	15	1.66	0.036169	GenMAPP
HSA04070_PHOSPHATIDYLINOSITOL_SIGNALING_SYSTEM	72	1.65	0.036979	KEGG
HSA00710_CARBON_FIXATION	20	1.65	0.037118	KEGG
TOB1PATHWAY	16	1.65	0.038963	BioCarta
SA_CASPASE_CASCADE	16	1.64	0.039033	SigmaAldrich
ATMPATHWAY	17	1.64	0.039992	BioCarta
SIG_INSULIN_RECEPTOR_PATHWAY_IN_CARDIAC_MYOCYTES	49	1.63	0.042054	SignalingAlliance
STRESSPATHWAY	25	1.63	0.042832	BioCarta
TYROSINE_METABOLISM	27	1.62	0.045745	GenMAPP
PENTOSE_PHOSPHATE_PATHWAY	21	1.61	0.046472	GenMAPP
GLUCONEOGENESIS	45	1.61	0.046872	GenMAPP
ST_PHOSPHOINOSITIDE_3_KINASE_PATHWAY	34	1.61	0.046950	STKE
HSA00030_PENTOSE_PHOSPHATE_PATHWAY	24	1.61	0.047101	KEGG
NFKBPATHWAY	23	1.61	0.047155	BioCarta
PGC1APATHWAY	22	1.60	0.047891	BioCarta
HSA04810_REGULATION_OF_ACTIN_CYTOSKELETON	193	1.60	0.048395	KEGG
CALCIUM_REGULATION_IN_CARDIAC_CELLS	135	1.60	0.048456	GenMAPP
GLYCOLYSIS	45	1.60	0.048470	GenMAPP
HSA00670_ONE_CARBON_POOL_BY_FOLATE	15	1.60	0.048629	KEGG
HSA03030_DNA_POLYMERASE	25	1.60	0.049184	KEGG
HSA05220_CHRONIC_MYELOID_LEUKEMIA	71	1.60	0.049601	KEGG

TCRPATHWAY	41	1.59	0.052076	BioCarta
HSA05040_HUNTINGTONS_DISEASE	28	1.58	0.055602	KEGG
HSA05010_ALZHEIMERS_DISEASE	26	1.57	0.056627	KEGG
HSA03050_PROTEASOME	22	1.57	0.057378	KEGG
PURINE_METABOLISM	105	1.57	0.057420	GenMAPP
HSA00350_TYROSINE_METABOLISM	53	1.57	0.057464	KEGG
NTHIPATHWAY	21	1.56	0.058542	BioCarta
HSA03020_RNA_POLYMERASE	19	1.55	0.062510	KEGG
CHEMICALPATHWAY	19	1.55	0.063491	BioCarta
CELL_MOTILITY	90	1.54	0.065929	GO
CELL_CYCLE_ARREST	26	1.54	0.067897	GO
ST_DICTYOSTELIUM_DISCOIDEUM_CAMP_CHEMOTAXIS_PATHWAY	31	1.54	0.067983	STKE
HSA04360_AXON_GUIDANCE	122	1.54	0.068070	KEGG
HSA04060_CYTOKINE_CYTOKINE_RECEPTOR_INTERACTION	218	1.53	0.070183	KEGG
HSA00770_PANTOTHENATE_AND_COA_BIOSYNTHESIS	15	1.53	0.070477	KEGG
HSA00150_ANDROGEN_AND_ESTROGEN_METABOLISM	37	1.52	0.073152	KEGG
HSA00500_STARCH_AND_SUCROSE_METABOLISM	62	1.52	0.074833	KEGG
HSA00562_INOSITOL_PHOSPHATE_METABOLISM	48	1.52	0.075486	KEGG
PHOSPHATIDYLINOSITOL_SIGNALING_SYSTEM	83	1.51	0.077818	GenMAPP
HSA05131_PATHOGENIC_ESCHERICHIA_COLI_INFECTION_EPEC	41	1.51	0.078091	KEGG
HSA05130_PATHOGENIC_ESCHERICHIA_COLI_INFECTION_EHEC	41	1.50	0.080456	KEGG
CELL_SURFACE_RECEPTOR_LINKED_SIGNAL_TRANSDUCTION	114	1.50	0.082527	GO
HSA04670_LEUKOCYTE_TRANSENDOTHELIAL_MIGRATION	106	1.49	0.084583	KEGG
HSA04370_VEGF_SIGNALING_PATHWAY	65	1.49	0.084828	KEGG
CK1PATHWAY	15	1.49	0.084950	BioCarta
HCMVPATHWAY	16	1.49	0.085251	BioCarta
CITRATE_CYCLE_TCA_CYCLE	17	1.49	0.086033	GenMAPP
HSA03320_PPAR_SIGNALING_PATHWAY	60	1.49	0.086310	KEGG
TRYPTOPHAN_METABOLISM	37	1.48	0.088132	GenMAPP
GLYCEROPHOSPHOLIPID_METABOLISM	46	1.48	0.090764	GenMAPP
SIG_CD40PATHWAYMAP	33	1.47	0.093259	SignalingAlliance
INTEGRINPATHWAY	34	1.47	0.094344	BioCarta
HSA00380_TRYPTOPHAN_METABOLISM	57	1.46	0.096855	KEGG
STARCH_AND_SUCROSE_METABOLISM	25	1.46	0.097067	GenMAPP
HSA05020_PARKINSONS_DISEASE	15	1.46	0.097843	KEGG
HSA04010_MAPK_SIGNALING_PATHWAY	240	1.46	0.099460	KEGG
ACTINYPATHWAY	18	1.45	0.102535	BioCarta
PHENYLALANINE_METABOLISM	21	1.45	0.103078	GenMAPP
ANDROGEN_AND_ESTROGEN_METABOLISM	16	1.45	0.105841	GenMAPP
STRIATED_MUSCLE_CONTRACTION	32	1.45	0.107297	GenMAPP
HSA00020_CITRATE_CYCLE	25	1.44	0.109879	KEGG
KERATINOCYTEPATHWAY	42	1.44	0.110023	BioCarta
HSA04620_TOLL_LIKE_RECEPTOR_SIGNALING_PATHWAY	96	1.44	0.110045	KEGG
COMPPATHWAY	15	1.44	0.110167	BioCarta
MAPKPATHWAY	83	1.44	0.111609	BioCarta
HSA04910_INSULIN_SIGNALING_PATHWAY	124	1.43	0.114292	KEGG
G_PROTEIN_SIGNALING	85	1.42	0.117796	GenMAPP
IL2RBPATHWAY	35	1.42	0.118876	BioCarta
SA_B_CELL_RECEPTOR_COMPLEXES	23	1.42	0.120299	SigmaAldrich

HSA00010_GLYCOLYSIS_AND_GLUCONEOGENESIS	53	1.42	0.122412	KEGG
GALACTOSE_METABOLISM	21	1.42	0.123317	GenMAPP
GLYCINE_SERINE_AND_THREONINE_METABOLISM	34	1.41	0.125160	GenMAPP
GHPATHWAY	23	1.40	0.134818	BioCarta
PROTEASOME	17	1.40	0.137335	GenMAPP
HSA00360_PHENYLALANINE_METABOLISM	29	1.39	0.137626	KEGG
OVARIAN_INFERTILITY_GENES	25	1.39	0.139295	GenMAPP
HSA00590_ARACHIDONIC_ACID_METABOLISM	39	1.39	0.140548	KEGG
HSA00564_GLYCEROPHOSPHOLIPID_METABOLISM	67	1.39	0.141876	KEGG
INOSITOL_PHOSPHATE_METABOLISM	23	1.39	0.143426	GenMAPP
FMLPPATHWAY	36	1.38	0.145654	BioCarta
MTORPATHWAY	22	1.38	0.148003	BioCarta
HSA04020_CALCIUM_SIGNALING_PATHWAY	161	1.37	0.150843	KEGG
P53HYPOXIAPATHWAY	17	1.37	0.152003	BioCarta
ARGININE_AND_PROLINE_METABOLISM	39	1.37	0.155112	GenMAPP
CELL_CYCLE_REGULATOR	21	1.36	0.158426	GO
HSA04940_TYPE_I_DIABETES_MELLITUS	21	1.36	0.158920	KEGG
HSA05210_COLORECTAL_CANCER	81	1.35	0.166522	KEGG
GPCRS_CLASS_B_SECRETIN_LIKE	19	1.35	0.166931	GO
UCALPAINPATHWAY	15	1.34	0.176800	BioCarta
GPCRDB_CLASS_B_SECRETIN_LIKE	19	1.33	0.184253	GenMAPP
VIPPATHWAY	25	1.32	0.187682	BioCarta
41BBPATHWAY	18	1.32	0.191055	BioCarta
IL12PATHWAY	20	1.32	0.191310	BioCarta
MEF2DPATHWAY	17	1.31	0.195941	BioCarta
ST_GA13_PATHWAY	33	1.31	0.198565	STKE
TH1TH2PATHWAY	15	1.30	0.211160	BioCarta
HSA05213_ENDOMETRIAL_CANCER	49	1.29	0.215954	KEGG
HSA04350_TGF_BETA_SIGNALING_PATHWAY	84	1.29	0.219104	KEGG
HSA00330_ARGININE_AND_PROLINE_METABOLISM	29	1.28	0.225394	KEGG
RAC1PATHWAY	22	1.28	0.227521	BioCarta
ST_WNT_CA2_CYCLIC_GMP_PATHWAY	18	1.27	0.231972	STKE
ERK5PATHWAY	15	1.27	0.233976	BioCarta
HSA05211_RENAL_CELL_CARCINOMA	67	1.27	0.239780	KEGG
HSA00790_FOLATE_BIOSYNTHESIS	37	1.26	0.242009	KEGG
HSA05030_AMYOTROPHIC_LATERAL_SCLEROSIS	17	1.26	0.248374	KEGG
HDACPATHWAY	28	1.25	0.249253	BioCarta
**IAA+ *vs.* IAA-**				
**Pathway**	**N**	**NES**	**FDR *q***	**Source**
INTRINSICPATHWAY	22	-2.17	0.004032	BioCarta
HSA04620_TOLL_LIKE_RECEPTOR_SIGNALING_PATHWAY	96	2.00	0.004060	KEGG
HSA04110_CELL_CYCLE	106	2.00	0.004208	KEGG
HSA04662_B_CELL_RECEPTOR_SIGNALING_PATHWAY	59	2.00	0.004226	KEGG
BLOOD_CLOTTING_CASCADE	19	-2.14	0.004525	GenMAPP
HIVNEFPATHWAY	53	1.97	0.004603	BioCarta
G1_TO_S_CELL_CYCLE_REACTOME	65	1.94	0.006759	GenMAPP
MAPKPATHWAY	83	1.93	0.007257	BioCarta
NFKBPATHWAY	23	1.93	0.007325	BioCarta

APOPTOSIS_GENMAPP	40	1.93	0.007348	GenMAPP
HSA04010_MAPK_SIGNALING_PATHWAY	240	1.91	0.007356	KEGG
NO2IL12PATHWAY	15	1.92	0.007410	BioCarta
HSA05222_SMALL_CELL_LUNG_CANCER	83	1.92	0.007464	KEGG
SA_B_CELL_RECEPTOR_COMPLEXES	23	1.90	0.008737	SigmaAldrich
HSA05130_PATHOGENIC_ESCHERICHIA_COLI_INFECTION_EHEC	41	1.88	0.011063	KEGG
HSA05131_PATHOGENIC_ESCHERICHIA_COLI_INFECTION_EPEC	41	1.88	0.011458	KEGG
TCRPATHWAY	41	1.86	0.013286	BioCarta
TIDPATHWAY	16	1.85	0.013433	BioCarta
IL12PATHWAY	20	1.85	0.013923	BioCarta
TOLLPATHWAY	34	1.84	0.014320	BioCarta
BCRPATHWAY	32	1.81	0.018133	BioCarta
HSA04930_TYPE_II_DIABETES_MELLITUS	42	1.81	0.018149	KEGG
APOPTOSIS	63	1.80	0.018300	GenMAPP
RELAPATHWAY	16	1.80	0.018432	BioCarta
SIG_PIP3_SIGNALING_IN_B_LYMPHOCYTES	33	1.79	0.019656	SignalingAlliance
HSA04660_T_CELL_RECEPTOR_SIGNALING_PATHWAY	90	1.79	0.019800	KEGG
HSA04650_NATURAL_KILLER_CELL_MEDIATED_CYTOTOXICITY	94	1.78	0.020025	KEGG
HSA04070_PHOSPHATIDYLINOSITOL_SIGNALING_SYSTEM	72	1.78	0.020201	KEGG
HSA04670_LEUKOCYTE_TRANSENDOTHELIAL_MIGRATION	106	1.78	0.020532	KEGG
ST_DIFFERENTIATION_PATHWAY_IN_PC12_CELLS	40	1.78	0.020593	STKE
HSA04210_APOPTOSIS	75	1.77	0.021354	KEGG
CELL_CYCLE	71	1.77	0.021411	GO
HSA01031_GLYCAN_STRUCTURES_BIOSYNTHESIS_2	56	1.77	0.021773	KEGG
ERKPATHWAY	28	1.76	0.023134	BioCarta
P38MAPKPATHWAY	37	1.76	0.023146	BioCarta
DNA_REPLICATION_REACTOME	40	1.76	0.023265	GenMAPP
HDACPATHWAY	28	1.75	0.023655	BioCarta
PHOSPHATIDYLINOSITOL_SIGNALING_SYSTEM	83	1.73	0.027528	GenMAPP
HSA04360_AXON_GUIDANCE	122	1.73	0.027840	KEGG
DEATHPATHWAY	30	1.72	0.028556	BioCarta
HSA05213_ENDOMETRIAL_CANCER	49	1.72	0.028903	KEGG
HSA04514_CELL_ADHESION_MOLECULES	105	1.72	0.029164	KEGG
KERATINOCYTEPATHWAY	42	1.72	0.029447	BioCarta
ST_B_CELL_ANTIGEN_RECEPTOR	37	1.71	0.030926	STKE
FMLPPATHWAY	36	1.71	0.030952	BioCarta
IGF1PATHWAY	19	1.69	0.032939	BioCarta
ST_JNK_MAPK_PATHWAY	38	1.70	0.033284	STKE
CCR3PATHWAY	20	1.68	0.035382	BioCarta
HSA04520_ADHERENS_JUNCTION	70	1.68	0.035893	KEGG
IL1RPATHWAY	32	1.68	0.036154	BioCarta
HSA00562_INOSITOL_PHOSPHATE_METABOLISM	48	1.68	0.036353	KEGG
HSA00600_SPHINGOLIPID_METABOLISM	33	1.68	0.036642	KEGG
RHOPATHWAY	29	1.67	0.037306	BioCarta
HSA00530_AMINOSUGARS_METABOLISM	27	1.67	0.038807	KEGG
HSA04060_CYTOKINE_CYTOKINE_RECEPTOR_INTERACTION	218	1.66	0.040099	KEGG
CASPASEPATHWAY	21	1.66	0.040112	BioCarta
HSA04810_REGULATION_OF_ACTIN_CYTOSKELETON	193	1.66	0.040485	KEGG
CELL_CYCLE_ARREST	26	1.65	0.042357	GO

HSA04640_HEMATOPOIETIC_CELL_LINEAGE	65	1.65	0.042469	KEGG
HSA00604_GLYCOSPHINGOLIPID_BIOSYNTHESIS_GANGLIOSERIES	15	1.65	0.042801	KEGG
HSA05218_MELANOMA	68	1.64	0.043442	KEGG
HSA05216_THYROID_CANCER	26	1.64	0.043758	KEGG
PAR1PATHWAY	19	1.64	0.043932	BioCarta
HSA05223_NON_SMALL_CELL_LUNG_CANCER	50	1.64	0.044199	KEGG
ERK5PATHWAY	15	1.64	0.045871	BioCarta
HSA04720_LONG_TERM_POTENTIATION	62	1.63	0.045886	KEGG
CSKPATHWAY	18	1.63	0.046347	BioCarta
AMIPATHWAY	18	1.63	0.047567	BioCarta
CELL_CYCLE_KEGG	79	1.62	0.048790	GenMAPP
NTHIPATHWAY	21	1.62	0.049388	BioCarta
TNFR2PATHWAY	18	1.62	0.049679	BioCarta
HSA04370_VEGF_SIGNALING_PATHWAY	65	1.62	0.050699	KEGG
SIG_INSULIN_RECEPTOR_PATHWAY_IN_CARDIAC_MYOCYTES	49	1.61	0.051398	SignalingAlliance
HSA00960_ALKALOID_BIOSYNTHESIS_II	20	1.61	0.051913	KEGG
GLYCOSPHINGOLIPID_METABOLISM	19	1.60	0.053678	GenMAPP
HSA04310_WNT_SIGNALING_PATHWAY	134	1.60	0.055435	KEGG
CTLA4PATHWAY	16	1.59	0.057089	BioCarta
CELL_ADHESION	166	1.59	0.057093	GO
HSA05215_PROSTATE_CANCER	82	1.59	0.057232	KEGG
HSA00190_OXIDATIVE_PHOSPHORYLATION	102	-1.84	0.060413	KEGG
ST_FAS_SIGNALING_PATHWAY	58	1.58	0.060995	STKE
METPATHWAY	34	1.58	0.062399	BioCarta
HSA04630_JAK_STAT_SIGNALING_PATHWAY	138	1.58	0.062423	KEGG
UBIQUINONE_BIOSYNTHESIS	15	-1.81	0.063986	GenMAPP
HSA01032_GLYCAN_STRUCTURES_DEGRADATION	27	1.57	0.064688	KEGG
ST_GA12_PATHWAY	22	1.57	0.067051	STKE
DCPATHWAY	21	1.56	0.068542	BioCarta
HSA04610_COMPLEMENT_AND_COAGULATION_CASCADES	62	-1.76	0.069260	KEGG
HSA05220_CHRONIC_MYELOID_LEUKEMIA	71	1.56	0.069812	KEGG
CERAMIDEPATHWAY	21	1.55	0.071550	BioCarta
SIG_BCR_SIGNALING_PATHWAY	46	1.55	0.072081	SignalingAlliance
BIOPEPTIDESPATHWAY	36	1.55	0.072510	BioCarta
SIG_CD40PATHWAYMAP	33	1.55	0.072797	SignalingAlliance
GPCRPATHWAY	32	1.54	0.074854	BioCarta
HSA00760_NICOTINATE_AND_NICOTINAMIDE_METABOLISM	21	1.54	0.074864	KEGG
HSA05210_COLORECTAL_CANCER	81	1.54	0.075104	KEGG
HSA04664_FC_EPSILON_RI_SIGNALING_PATHWAY	73	1.54	0.075527	KEGG
APOPTOSIS_KEGG	46	1.54	0.076461	GenMAPP
HSA05212_PANCREATIC_CANCER	71	1.54	0.077011	KEGG
CXCR4PATHWAY	22	1.52	0.082554	BioCarta
CELL_MOTILITY	90	1.52	0.085724	GO
CELL_PROLIFERATION	187	1.51	0.088046	GO
MITOCHONDRIAPATHWAY	19	1.51	0.088192	BioCarta
HSA04920_ADIPOCYTOKINE_SIGNALING_PATHWAY	71	1.50	0.090091	KEGG
HSA00670_ONE_CARBON_POOL_BY_FOLATE	15	1.50	0.091131	KEGG
HSA05214_GLIOMA	58	1.50	0.091553	KEGG
GHPATHWAY	23	1.50	0.091999	BioCarta

HSA00531_GLYCOSAMINOGLYCAN_DEGRADATION	15	1.50	0.092406	KEGG
UBIQUITIN_MEDIATED_PROTEOLYSIS	23	1.50	0.092656	GenMAPP
G2PATHWAY	21	1.50	0.092732	BioCarta
HSA04115_P53_SIGNALING_PATHWAY	62	1.50	0.092779	KEGG
IL7PATHWAY	16	1.49	0.093420	BioCarta
STRESSPATHWAY	25	1.49	0.093458	BioCarta
CELL_CYCLE_CHECKPOINT	22	1.49	0.093617	GO
ST_P38_MAPK_PATHWAY	36	1.49	0.095418	STKE
CHEMICALPATHWAY	19	1.48	0.097960	BioCarta
ST_TUMOR_NECROSIS_FACTOR_PATHWAY	29	1.48	0.101127	STKE
CCR5PATHWAY	17	1.46	0.107582	BioCarta
SIG_PIP3_SIGNALING_IN_CARDIAC_MYOCTES	63	1.46	0.108418	SignalingAlliance
HSA05040_HUNTINGTONS_DISEASE	28	1.46	0.108542	KEGG
HCMVPATHWAY	16	1.46	0.108937	BioCarta
HSA03022_BASAL_TRANSCRIPTION_FACTORS	30	1.46	0.109079	KEGG
WNTPATHWAY	24	1.46	0.109458	BioCarta
HSA04540_GAP_JUNCTION	81	1.46	0.110080	KEGG
MRNA_PROCESSING_REACTOME	102	1.46	0.110230	GenMAPP
HSA04530_TIGHT_JUNCTION	116	1.45	0.114523	KEGG
CELLCYCLEPATHWAY	22	1.44	0.114680	BioCarta
NGFPATHWAY	17	1.42	0.127117	BioCarta
HSA04730_LONG_TERM_DEPRESSION	70	1.42	0.127367	KEGG
GSK3PATHWAY	26	1.42	0.131351	BioCarta
TH1TH2PATHWAY	15	1.41	0.133904	BioCarta
EPOPATHWAY	18	1.41	0.137291	BioCarta
HSA04512_ECM_RECEPTOR_INTERACTION	83	1.41	0.138602	KEGG
HSA04510_FOCAL_ADHESION	185	1.40	0.139811	KEGG
HSA01510_NEURODEGENERATIVE_DISEASES	36	1.40	0.139983	KEGG
CALCIUM_REGULATION_IN_CARDIAC_CELLS	135	1.40	0.143249	GenMAPP
ST_PHOSPHOINOSITIDE_3_KINASE_PATHWAY	34	1.40	0.144404	STKE
ST_T_CELL_SIGNAL_TRANSDUCTION	44	1.39	0.144666	STKE
MPRPATHWAY	20	1.39	0.144762	BioCarta
GO_ROS	27	1.39	0.144939	GO
ATMPATHWAY	17	1.39	0.147567	BioCarta
HSA05010_ALZHEIMERS_DISEASE	26	1.39	0.147607	KEGG
HYPERTROPHY_MODEL	16	1.39	0.147778	GenMAPP
HSA04916_MELANOGENESIS	92	1.38	0.149708	KEGG
TNFR1PATHWAY	27	1.38	0.150405	BioCarta
HSA05221_ACUTE_MYELOID_LEUKEMIA	50	1.38	0.150517	KEGG
GLEEVECPATHWAY	21	1.38	0.151518	BioCarta
P53PATHWAY	15	1.38	0.151563	BioCarta
NKTPATHWAY	27	1.38	0.151677	BioCarta
HSA03050_PROTEASOME	22	1.38	0.151795	KEGG
HSA05217_BASAL_CELL_CARCINOMA	53	1.38	0.151799	KEGG
INSULINPATHWAY	19	1.38	0.153173	BioCarta
HSA04912_GNRH_SIGNALING_PATHWAY	90	1.38	0.153290	KEGG
HSA04150_MTOR_SIGNALING_PATHWAY	44	1.37	0.154920	KEGG
PGC1APATHWAY	22	1.37	0.157509	BioCarta
HSA04910_INSULIN_SIGNALING_PATHWAY	124	1.37	0.158019	KEGG

P53HYPOXIAPATHWAY	17	1.37	0.158257	BioCarta
SA_TRKA_RECEPTOR	15	1.37	0.158920	SigmaAldrich
EGFPATHWAY	25	1.36	0.159636	BioCarta
HSA00450_SELENOAMINO_ACID_METABOLISM	25	1.36	0.159669	KEGG
WNT_SIGNALING	58	1.36	0.160176	GEArray
VIPPATHWAY	25	1.35	0.168961	BioCarta
NFATPATHWAY	49	1.35	0.169361	BioCarta
HSP27PATHWAY	15	1.35	0.169991	BioCarta
TPOPATHWAY	20	1.35	0.171205	BioCarta
GPCRS_CLASS_A_RHODOPSIN_LIKE	123	1.34	0.177967	GO
FCER1PATHWAY	36	1.34	0.178345	BioCarta
HSA00350_TYROSINE_METABOLISM	53	1.33	0.180485	KEGG
CALCINEURINPATHWAY	18	1.33	0.184247	BioCarta
AT1RPATHWAY	30	1.33	0.186365	BioCarta
CELL_CYCLE_REGULATOR	21	1.32	0.187074	GO
HSA04130_SNARE_INTERACTIONS_IN_VESICULAR_TRANSPORT	30	1.32	0.187637	KEGG
MEF2DPATHWAY	17	1.32	0.188420	BioCarta
CELL_ADHESION_MOLECULE_ACTIVITY	104	1.32	0.190142	GO
VALINE_LEUCINE_AND_ISOLEUCINE_DEGRADATION	34	-1.61	0.191992	GenMAPP
CALCINEURIN_NF_AT_SIGNALING	86	1.32	0.192266	GEArray
BREAST_CANCER_ESTROGEN_SIGNALING	83	1.31	0.194252	GEArray
HSA04612_ANTIGEN_PROCESSING_AND_PRESENTATION	42	1.31	0.196557	KEGG
SIG_IL4RECEPTOR_IN_B_LYPHOCYTES	26	1.31	0.198466	SignalingAlliance
FASPATHWAY	26	1.30	0.201717	BioCarta
CK1PATHWAY	15	1.30	0.203675	BioCarta
HSA04012_ERBB_SIGNALING_PATHWAY	81	1.30	0.204179	KEGG
PROTEASOME	17	1.30	0.204969	GenMAPP
G1PATHWAY	23	1.29	0.212488	BioCarta
PROPANOATE_METABOLISM	28	-1.55	0.220346	GenMAPP
STRIATED_MUSCLE_CONTRACTION	32	1.28	0.223800	GenMAPP
HSA00602_GLYCOSPHINGOLIPID_BIOSYNTHESIS_NEO_LACTOSERIES	17	1.28	0.223931	KEGG
COMPPATHWAY	15	1.28	0.224784	BioCarta
HSA00860_PORPHYRIN_AND_CHLOROPHYLL_METABOLISM	29	1.27	0.232726	KEGG
PDGFPATHWAY	25	1.27	0.233463	BioCarta
NDKDYNAMINPATHWAY	19	1.27	0.233653	BioCarta
HSA00532_CHONDROITIN_SULFATE_BIOSYNTHESIS	17	1.27	0.235585	KEGG
PORPHYRIN_AND_CHLOROPHYLL_METABOLISM	16	1.27	0.237722	GenMAPP
INOSITOL_PHOSPHATE_METABOLISM	23	1.26	0.239106	GenMAPP
RACCYCDPATHWAY	21	1.26	0.239858	BioCarta
MONOAMINE_GPCRS	30	1.26	0.240071	GenMAPP
HSA04020_CALCIUM_SIGNALING_PATHWAY	161	1.26	0.242739	KEGG
ST_G_ALPHA_I_PATHWAY	34	1.26	0.242814	STKE
HSA04320_DORSO_VENTRAL_AXIS_FORMATION	26	1.26	0.243666	KEGG
PYK2PATHWAY	27	1.25	0.248223	BioCarta
**IAA+ non-progressors *vs.* other**				
**Pathway**	**N**	**NES**	**FDR *q***	**Source**
HSA04662_B_CELL_RECEPTOR_SIGNALING_PATHWAY	59	2.52	0.000000	KEGG
NFKBPATHWAY	23	2.34	0.000000	BioCarta

ST_TUMOR_NECROSIS_FACTOR_PATHWAY	29	2.24	0.000000	STKE
HSA04010_MAPK_SIGNALING_PATHWAY	240	2.22	0.000150	KEGG
HSA04210_APOPTOSIS	75	2.16	0.000177	KEGG
HSA04620_TOLL_LIKE_RECEPTOR_SIGNALING_PATHWAY	96	2.16	0.000183	KEGG
ST_JNK_MAPK_PATHWAY	38	2.17	0.000197	STKE
TOLLPATHWAY	34	2.18	0.000222	BioCarta
MAPKPATHWAY	83	2.14	0.000317	BioCarta
HIVNEFPATHWAY	53	2.13	0.000328	BioCarta
HSA04510_FOCAL_ADHESION	185	2.11	0.000329	KEGG
HSA05220_CHRONIC_MYELOID_LEUKEMIA	71	2.11	0.000345	KEGG
HSA05214_GLIOMA	58	2.09	0.000524	KEGG
APOPTOSIS_GENMAPP	40	2.08	0.000531	GenMAPP
HSA05218_MELANOMA	68	2.07	0.000697	KEGG
HSA04660_T_CELL_RECEPTOR_SIGNALING_PATHWAY	90	2.06	0.000709	KEGG
APOPTOSIS	63	2.06	0.000742	GenMAPP
ST_INTEGRIN_SIGNALING_PATHWAY	78	2.06	0.000774	STKE
SIG_BCR_SIGNALING_PATHWAY	46	2.05	0.000805	SignalingAlliance
ST_P38_MAPK_PATHWAY	36	2.05	0.000843	STKE
TIDPATHWAY	16	2.05	0.000845	BioCarta
HSA04360_AXON_GUIDANCE	122	2.04	0.000899	KEGG
IL1RPATHWAY	32	2.04	0.001026	BioCarta
BCRPATHWAY	32	2.03	0.001035	BioCarta
HSA05223_NON_SMALL_CELL_LUNG_CANCER	50	2.00	0.001484	KEGG
ST_B_CELL_ANTIGEN_RECEPTOR	37	2.00	0.001497	STKE
HSA04670_LEUKOCYTE_TRANSENDOTHELIAL_MIGRATION	106	2.00	0.001510	KEGG
HSA05212_PANCREATIC_CANCER	71	2.00	0.001512	KEGG
HSA04810_REGULATION_OF_ACTIN_CYTOSKELETON	193	1.99	0.001678	KEGG
HSA05211_RENAL_CELL_CARCINOMA	67	1.99	0.001681	KEGG
NTHIPATHWAY	21	1.98	0.001919	BioCarta
COMPPATHWAY	15	1.96	0.002314	BioCarta
RELAPATHWAY	16	1.96	0.002318	BioCarta
P38MAPKPATHWAY	37	1.96	0.002501	BioCarta
INTEGRIN_MEDIATED_CELL_ADHESION_KEGG	90	1.95	0.002570	GenMAPP
STRESSPATHWAY	25	1.95	0.002678	BioCarta
IL6PATHWAY	19	1.95	0.002717	BioCarta
HSA04110_CELL_CYCLE	106	1.92	0.003277	KEGG
HSA04930_TYPE_II_DIABETES_MELLITUS	42	1.93	0.003317	KEGG
APOPTOSIS_KEGG	46	1.93	0.003334	GenMAPP
SIG_CD40PATHWAYMAP	33	1.93	0.003338	SignalingAlliance
HSA05213_ENDOMETRIAL_CANCER	49	1.92	0.003412	KEGG
SA_B_CELL_RECEPTOR_COMPLEXES	23	1.90	0.003574	SigmaAldrich
HSA05210_COLORECTAL_CANCER	81	1.89	0.003975	KEGG
HSA05222_SMALL_CELL_LUNG_CANCER	83	1.88	0.004517	KEGG
HSA00190_OXIDATIVE_PHOSPHORYLATION	102	-2.14	0.004580	KEGG
TNFR1PATHWAY	27	1.87	0.004731	BioCarta
FCER1PATHWAY	36	1.87	0.004820	BioCarta
PDGFPATHWAY	25	1.86	0.005250	BioCarta
IL12PATHWAY	20	1.86	0.005396	BioCarta
SA_PTEN_PATHWAY	16	1.85	0.005938	SigmaAldrich

HSA04514_CELL_ADHESION_MOLECULES	105	1.85	0.005948	KEGG
ST_INTERLEUKIN_4_PATHWAY	23	1.84	0.006384	STKE
KERATINOCYTEPATHWAY	42	1.84	0.006403	BioCarta
HSA04512_ECM_RECEPTOR_INTERACTION	83	1.83	0.006832	KEGG
SA_TRKA_RECEPTOR	15	1.83	0.006914	SigmaAldrich
ERKPATHWAY	28	1.83	0.007152	BioCarta
CELL_CYCLE	71	1.80	0.008602	GO
METPATHWAY	34	1.79	0.009319	BioCarta
MRNA_PROCESSING_REACTOME	102	1.79	0.009784	GenMAPP
EGFPATHWAY	25	1.78	0.010251	BioCarta
TNFR2PATHWAY	18	1.78	0.010610	BioCarta
HSA04370_VEGF_SIGNALING_PATHWAY	65	1.78	0.010649	KEGG
SIG_CHEMOTAXIS	44	1.77	0.011176	SignalingAlliance
HSA04520_ADHERENS_JUNCTION	70	1.77	0.011244	KEGG
ST_DIFFERENTIATION_PATHWAY_IN_PC12_CELLS	40	1.76	0.012420	STKE
HSA04650_NATURAL_KILLER_CELL_MEDIATED_CYTOTOXICITY	94	1.76	0.012555	KEGG
HSA04664_FC_EPSILON_RI_SIGNALING_PATHWAY	73	1.75	0.012855	KEGG
DEATHPATHWAY	30	1.75	0.012876	BioCarta
CELL_GROWTH_AND_OR_MAINTENANCE	58	1.75	0.012905	GO
HSA04012_ERBB_SIGNALING_PATHWAY	81	1.75	0.013063	KEGG
HSA04060_CYTOKINE_CYTOKINE_RECEPTOR_INTERACTION	218	1.75	0.013460	KEGG
CERAMIDEPATHWAY	21	1.75	0.013476	BioCarta
ECMPATHWAY	20	1.73	0.015894	BioCarta
CELL_ADHESION	166	1.73	0.016246	GO
SIG_PIP3_SIGNALING_IN_B_LYMPHOCYTES	33	1.72	0.016639	SignalingAlliance
HSA05215_PROSTATE_CANCER	82	1.71	0.017523	KEGG
HSA04310_WNT_SIGNALING_PATHWAY	134	1.71	0.017545	KEGG
CELL_CYCLE_KEGG	79	1.71	0.017550	GenMAPP
HSA05131_PATHOGENIC_ESCHERICHIA_COLI_INFECTION_EPEC	41	1.71	0.017573	KEGG
NO2IL12PATHWAY	15	1.71	0.017594	BioCarta
STRIATED_MUSCLE_CONTRACTION	32	1.70	0.018226	GenMAPP
HSA04910_INSULIN_SIGNALING_PATHWAY	124	1.70	0.018233	KEGG
TCRPATHWAY	41	1.70	0.018377	BioCarta
HSA05221_ACUTE_MYELOID_LEUKEMIA	50	1.68	0.021013	KEGG
RAC1PATHWAY	22	1.68	0.021239	BioCarta
ST_FAS_SIGNALING_PATHWAY	58	1.68	0.021316	STKE
PHOSPHATIDYLINOSITOL_SIGNALING_SYSTEM	83	1.68	0.021407	GenMAPP
CREBPATHWAY	24	1.67	0.021899	BioCarta
HSA05130_PATHOGENIC_ESCHERICHIA_COLI_INFECTION_EHEC	41	1.67	0.022014	KEGG
AMIPATHWAY	18	1.67	0.022937	BioCarta
CSKPATHWAY	18	1.66	0.024045	BioCarta
HSA04920_ADIPOCYTOKINE_SIGNALING_PATHWAY	71	1.66	0.024251	KEGG
HSA05216_THYROID_CANCER	26	1.65	0.025702	KEGG
PTENPATHWAY	16	1.65	0.025819	BioCarta
SIG_INSULIN_RECEPTOR_PATHWAY_IN_CARDIAC_MYOCYTES	49	1.64	0.028608	SignalingAlliance
ERK5PATHWAY	15	1.64	0.028636	BioCarta
HSA04912_GNRH_SIGNALING_PATHWAY	90	1.63	0.028964	KEGG
FMLPPATHWAY	36	1.63	0.030784	BioCarta
RHOPATHWAY	29	1.62	0.031839	BioCarta

ST_GA13_PATHWAY	33	1.61	0.033382	STKE
CELL_CYCLE_CHECKPOINT	22	1.61	0.033461	GO
DNA_REPLICATION_REACTOME	40	1.61	0.034040	GenMAPP
HSA04540_GAP_JUNCTION	81	1.61	0.034108	KEGG
GLYCOSPHINGOLIPID_METABOLISM	19	1.61	0.034281	GenMAPP
DCPATHWAY	21	1.61	0.034975	BioCarta
HCMVPATHWAY	16	1.60	0.035464	BioCarta
CK1PATHWAY	15	1.60	0.037143	BioCarta
HSA00604_GLYCOSPHINGOLIPID_BIOSYNTHESIS_GANGLIOSERIES	15	1.60	0.037222	KEGG
HSA04530_TIGHT_JUNCTION	116	1.60	0.037279	KEGG
CALCINEURINPATHWAY	18	1.60	0.037552	BioCarta
HSA00562_INOSITOL_PHOSPHATE_METABOLISM	48	1.60	0.037588	KEGG
HSA04070_PHOSPHATIDYLINOSITOL_SIGNALING_SYSTEM	72	1.60	0.037639	KEGG
HYPERTROPHY_MODEL	16	1.59	0.039159	GenMAPP
VIPPATHWAY	25	1.59	0.039264	BioCarta
HSA05010_ALZHEIMERS_DISEASE	26	1.59	0.039327	KEGG
UBIQUINONE_BIOSYNTHESIS	15	-1.88	0.039765	GenMAPP
CELL_PROLIFERATION	187	1.59	0.039878	GO
IL7PATHWAY	16	1.58	0.041261	BioCarta
G1_TO_S_CELL_CYCLE_REACTOME	65	1.58	0.041463	GenMAPP
ST_GA12_PATHWAY	22	1.58	0.041736	STKE
ST_G_ALPHA_I_PATHWAY	34	1.57	0.042083	STKE
HSA01510_NEURODEGENERATIVE_DISEASES	36	1.57	0.042227	KEGG
EPOPATHWAY	18	1.57	0.042990	BioCarta
FASPATHWAY	26	1.57	0.043502	BioCarta
HSA01032_GLYCAN_STRUCTURES_DEGRADATION	27	1.57	0.044056	KEGG
G1PATHWAY	23	1.56	0.044691	BioCarta
CYTOKINEPATHWAY	19	1.56	0.044862	BioCarta
BIOPEPTIDESPATHWAY	36	1.55	0.047203	BioCarta
ST_ERK1_ERK2_MAPK_PATHWAY	29	1.55	0.047581	STKE
CELL_ADHESION_RECEPTOR_ACTIVITY	32	1.55	0.048590	GO
HDACPATHWAY	28	1.54	0.051577	BioCarta
CALCINEURIN_NF_AT_SIGNALING	86	1.54	0.052034	GEArray
TPOPATHWAY	20	1.54	0.052081	BioCarta
SIG_PIP3_SIGNALING_IN_CARDIAC_MYOCTES	63	1.53	0.053288	SignalingAlliance
WNT_SIGNALING	58	1.53	0.054353	GEArray
HSA02010_ABC_TRANSPORTERS_GENERAL	41	1.53	0.055142	KEGG
PAR1PATHWAY	19	1.53	0.055254	BioCarta
CELL_MOTILITY	90	1.52	0.055995	GO
CELL_CYCLE_ARREST	26	1.52	0.056584	GO
HSA04640_HEMATOPOIETIC_CELL_LINEAGE	65	1.52	0.057354	KEGG
CALCIUM_REGULATION_IN_CARDIAC_CELLS	135	1.52	0.057648	GenMAPP
ST_T_CELL_SIGNAL_TRANSDUCTION	44	1.51	0.058764	STKE
ST_GRANULE_CELL_SURVIVAL_PATHWAY	25	1.51	0.060912	STKE
CHEMICALPATHWAY	19	1.51	0.061006	BioCarta
RACCYCDPATHWAY	21	1.51	0.061753	BioCarta
HSA04630_JAK_STAT_SIGNALING_PATHWAY	138	1.51	0.062109	KEGG
AT1RPATHWAY	30	1.50	0.062660	BioCarta
HSA05219_BLADDER_CANCER	37	1.50	0.062819	KEGG

HSA00531_GLYCOSAMINOGLYCAN_DEGRADATION	15	1.50	0.063084	KEGG
INSULINPATHWAY	19	1.50	0.063253	BioCarta
G_PROTEIN_SIGNALING	85	1.50	0.065147	GenMAPP
NGFPATHWAY	17	1.49	0.067308	BioCarta
CELL_ADHESION_MOLECULE_ACTIVITY	104	1.49	0.068848	GO
P53PATHWAY	15	1.48	0.072386	BioCarta
VALINE_LEUCINE_AND_ISOLEUCINE_DEGRADATION	34	-1.79	0.073041	GenMAPP
HSA04916_MELANOGENESIS	92	1.47	0.074431	KEGG
SIG_IL4RECEPTOR_IN_B_LYPHOCYTES	26	1.46	0.077564	SignalingAlliance
MEF2DPATHWAY	17	1.46	0.078840	BioCarta
HSA04150_MTOR_SIGNALING_PATHWAY	44	1.46	0.078904	KEGG
HSA01031_GLYCAN_STRUCTURES_BIOSYNTHESIS_2	56	1.46	0.079461	KEGG
OXIDATIVE_PHOSPHORYLATION	56	-1.76	0.080640	GenMAPP
ST_PHOSPHOINOSITIDE_3_KINASE_PATHWAY	34	1.44	0.085895	STKE
HSA04720_LONG_TERM_POTENTIATION	62	1.44	0.086491	KEGG
HSA04115_P53_SIGNALING_PATHWAY	62	1.44	0.086917	KEGG
MCALPAINPATHWAY	22	1.44	0.087038	BioCarta
EICOSANOID_SYNTHESIS	16	1.43	0.089638	GenMAPP
EDG1PATHWAY	22	1.43	0.090274	BioCarta
HSA00600_SPHINGOLIPID_METABOLISM	33	1.43	0.092689	KEGG
SIG_REGULATION_OF_THE_ACTIN_CYTOSKELETON_BY_RHO_GTPASES	34	1.43	0.093088	SignalingAlliance
MPRPATHWAY	20	1.42	0.094461	BioCarta
GATA3PATHWAY	15	1.43	0.094475	BioCarta
HSA05217_BASAL_CELL_CARCINOMA	53	1.42	0.094647	KEGG
HSA01030_GLYCAN_STRUCTURES_BIOSYNTHESIS_1	103	1.42	0.095837	KEGG
SMOOTH_MUSCLE_CONTRACTION	137	1.42	0.097681	GenMAPP
INFLAMPATHWAY	26	1.42	0.097768	BioCarta
IGF1PATHWAY	19	1.42	0.099272	BioCarta
INTEGRINPATHWAY	34	1.42	0.099645	BioCarta
CXCR4PATHWAY	22	1.41	0.100160	BioCarta
GHPATHWAY	23	1.41	0.103306	BioCarta
HSA04340_HEDGEHOG_SIGNALING_PATHWAY	54	1.40	0.108732	KEGG
PTDINSPATHWAY	21	1.40	0.110692	BioCarta
CASPASEPATHWAY	21	1.39	0.111524	BioCarta
BREAST_CANCER_ESTROGEN_SIGNALING	83	1.39	0.111656	GEArray
HSA04610_COMPLEMENT_AND_COAGULATION_CASCADES	62	1.39	0.112322	KEGG
ST_MYOCYTE_AD_PATHWAY	23	1.39	0.112513	STKE
PYK2PATHWAY	27	1.39	0.112551	BioCarta
HSA00310_LYSINE_DEGRADATION	45	1.39	0.112729	KEGG
CTLA4PATHWAY	16	1.39	0.114961	BioCarta
CELL_SURFACE_RECEPTOR_LINKED_SIGNAL_TRANSDUCTION	114	1.38	0.120103	GO
HSP27PATHWAY	15	1.38	0.120409	BioCarta
HSA04120_UBIQUITIN_MEDIATED_PROTEOLYSIS	36	1.37	0.122182	KEGG
HSA05120_EPITHELIAL_CELL_SIGNALING_IN_HELICOBACTER_PYLORI_INFECTION	64	1.37	0.122841	KEGG
HISTIDINE_METABOLISM	25	1.37	0.124207	GenMAPP
HSA04350_TGF_BETA_SIGNALING_PATHWAY	84	1.37	0.125073	KEGG
GPCRPATHWAY	32	1.37	0.127248	BioCarta
KREBS_TCA_CYCLE	28	-1.66	0.129344	GenMAPP
BADPATHWAY	20	1.36	0.132345	BioCarta

MITOCHONDRIAPATHWAY	19	1.34	0.144933	BioCarta
CELLCYCLEPATHWAY	22	1.34	0.144983	BioCarta
NFATPATHWAY	49	1.34	0.145035	BioCarta
EIF4PATHWAY	22	1.34	0.148116	BioCarta
MITOCHONDRIAL_FATTY_ACID_BETAOXIDATION	15	-1.61	0.152160	GenMAPP
CITRATE_CYCLE_TCA_CYCLE	17	-1.61	0.161203	GenMAPP
HSA05040_HUNTINGTONS_DISEASE	28	1.32	0.163369	KEGG
HSA03010_RIBOSOME	55	-1.57	0.163501	KEGG
HSA04130_SNARE_INTERACTIONS_IN_VESICULAR_TRANSPORT	30	1.32	0.164244	KEGG
GO_ROS	27	1.31	0.165600	GO
P53HYPOXIAPATHWAY	17	1.31	0.166857	BioCarta
HSA00360_PHENYLALANINE_METABOLISM	29	1.31	0.167978	KEGG
GLEEVECPATHWAY	21	1.30	0.172155	BioCarta
HSA04330_NOTCH_SIGNALING_PATHWAY	40	1.30	0.178129	KEGG
GSK3PATHWAY	26	1.30	0.178696	BioCarta
RASPATHWAY	20	1.29	0.180500	BioCarta
HSA00532_CHONDROITIN_SULFATE_BIOSYNTHESIS	17	1.29	0.181143	KEGG
G2PATHWAY	21	1.29	0.184482	BioCarta
HSA00740_RIBOFLAVIN_METABOLISM	15	1.28	0.188365	KEGG
CCR5PATHWAY	17	1.28	0.190858	BioCarta
41BBPATHWAY	18	1.28	0.194529	BioCarta
HSA00480_GLUTATHIONE_METABOLISM	34	-1.45	0.196112	KEGG
HSA04020_CALCIUM_SIGNALING_PATHWAY	161	1.28	0.196113	KEGG
HSA00280_VALINE_LEUCINE_AND_ISOLEUCINE_DEGRADATION	40	-1.46	0.196296	KEGG
ST_GAQ_PATHWAY	26	1.27	0.196744	STKE
NKTPATHWAY	27	1.27	0.200150	BioCarta
ST_WNT_BETA_CATENIN_PATHWAY	31	1.27	0.201906	STKE
CELL_CYCLE_REGULATOR	21	1.26	0.207376	GO
HSA00361_GAMMA_HEXACHLOROCYCLOHEXANE_DEGRADATION	16	1.26	0.209131	KEGG
HSA00340_HISTIDINE_METABOLISM	40	1.26	0.211225	KEGG
WNTPATHWAY	24	1.26	0.213279	BioCarta
PROPANOATE_METABOLISM	28	-1.43	0.213837	GenMAPP
HSA00071_FATTY_ACID_METABOLISM	39	-1.42	0.215951	KEGG
HSA00100_BIOSYNTHESIS_OF_STEROIDS	21	1.25	0.216033	KEGG
HSA00760_NICOTINATE_AND_NICOTINAMIDE_METABOLISM	21	1.24	0.224446	KEGG
BILE_ACID_BIOSYNTHESIS	21	-1.48	0.227176	GenMAPP
CCR3PATHWAY	20	1.24	0.231831	BioCarta
HSA00350_TYROSINE_METABOLISM	53	1.23	0.235183	KEGG
ETSPATHWAY	15	1.23	0.243830	BioCarta
ATMPATHWAY	17	1.22	0.247796	BioCarta
