# Supplementary material for: Metabolic Regulation in Progression to Autoimmune Diabetes
Source: PLoS Comput Biol. 2011 Oct 27;7(10):e1002257. doi: 10.1371/journal.pcbi.1002257 (PMC3203065; doi:10.1371/journal.pcbi.1002257)
Supplement: Table S2 — Leading edge genes in selected islet pathways. Leading edge genes for selected upregulated pathways (diabetes progressors vs. non-progressors in female NOD mice; Table 1 and Table S1) derived from Gene Set Enrichment Analysis [20] of islet gene expression data. NES, normalized enrichment score; FDR q, False Discovery Rate q-value [15]; Probe ID, Illumina Mouse WG-6 Expression BeadChips probe ID; Fold, fold change progressors vs. non-progressors; P-value (t statistic); Gene, common gene name. (PDF) [file pcbi.1002257.s005.pdf]

HIVNEFPATHWAY (NES = 2.32; FDR $q < 0.000001$)			
**Probe Id**	**Fold**	***P*-value**	**Gene**
ILMN_2648548	1.15	0.0427	CASP7
ILMN_1243876	1.37	0.0563	CASP8
ILMN_1227324	1.08	0.0786	PRKDC
ILMN_2582933	1.09	0.0803	TNFRSF6
ILMN_2789948	1.92	0.0819	ARHGDIB
ILMN_1217061	0.89	0.0865	CASP9
ILMN_1249021	1.22	0.0881	BCL2
ILMN_2505100	1.17	0.1028	TRADD
ILMN_2679386	0.83	0.1061	GSN
ILMN_2682162	1.19	0.1088	BCL2
ILMN_2592476	1.28	0.1107	NFKB1
ILMN_1233313	1.07	0.1191	TRADD
ILMN_2916035	1.34	0.1413	LMNB1
ILMN_3097868	1.15	0.1440	CASP8
ILMN_1230586	1.46	0.1477	TRAF1
ILMN_3161878	1.26	0.1601	BID
ILMN_1250774	1.06	0.1629	MDM2
ILMN_2626939	1.20	0.1751	DAXX
ILMN_2669404	1.10	0.1848	LMNB2
ILMN_2665050	1.29	0.1988	MAP3K14
ILMN_1213604	0.93	0.2135	ARHGDIB
ILMN_1218605	1.07	0.2317	NFKBIA
ILMN_2735625	1.13	0.2327	NUMA1
ILMN_2576736	1.23	0.2388	NFKB1
ILMN_2679619	1.07	0.2394	RB1
ILMN_2641705	1.07	0.2463	CDC2L1
ILMN_1223126	1.10	0.2486	FADD
ILMN_2637266	1.23	0.2525	CASP2
ILMN_3143577	1.17	0.2550	APAF1
ILMN_1253947	1.16	0.2638	TRAF1
ILMN_2605645	1.11	0.2703	GSN
ILMN_2645390	1.09	0.2773	PAK2
ILMN_1228387	1.06	0.2811	CASP2
ILMN_2466335	1.17	0.2827	APAF1
ILMN_2768200	1.03	0.2860	MAP2K7
ILMN_3006611	1.09	0.2872	MAP3K1
ILMN_1250564	1.06	0.2873	CYCS
ILMN_2485839	1.14	0.2922	TNFRSF1B
ILMN_2674731	1.04	0.2970	CASP9
ILMN_2761689	1.06	0.3063	PSEN1
ILMN_2486186	1.03	0.3131	TNFRSF1B
ILMN_3155380	1.04	0.3131	CYCS
ILMN_1241980	0.95	0.4009	CDC2L1
ILMN_2861644	1.09	0.4034	RB1
ILMN_3028981	0.96	0.4371	MAP2K7
ILMN_3001914	1.13	0.4435	NFKBIA
ILMN_1215465	1.05	0.4456	PAK2

ILMN_2720229	1.03	0.4763	LMNB1
ILMN_2662509	1.04	0.4840	FADD
ILMN_1228561	1.06	0.5004	APAF1
ILMN_2743517	0.97	0.5033	PRKDC
ILMN_1214811	0.95	0.5442	PSEN1
ILMN_3076459	0.98	0.6018	CYCS
ILMN_2757287	1.03	0.6054	BID
ILMN_3102163	1.03	0.6255	MAP2K7
ILMN_1257180	0.98	0.6397	PSEN1
ILMN_2874409	0.98	0.7002	FADD
ILMN_1229882	1.01	0.7024	MDM2
ILMN_2665625	0.98	0.7213	FADD
ILMN_2850753	0.98	0.7425	MDM2
ILMN_1255180	0.99	0.7515	CASP9
ILMN_1255973	1.01	0.7642	TNFRSF1B
ILMN_2614380	1.04	0.7985	MAP3K1
ILMN_2687249	0.99	0.8046	PSEN1
ILMN_2758139	1.01	0.8214	CASP9
ILMN_1241869	1.00	0.8982	MAP3K14
ILMN_3035785	1.00	0.9329	MAP2K7
**CTLA4PATHWAY (NES = 2.02; FDR *q* = 0.0019)**			
**Probe ID**	**Fold**	***P*-value**	**Gene**
ILMN_2784580	2.26	0.0347	CD3E
ILMN_2697415	2.51	0.0360	CD3D
ILMN_1238847	2.43	0.0426	CD3E
ILMN_2651137	2.23	0.0506	LCK
ILMN_2722784	2.43	0.0509	CD3G
ILMN_2613422	1.93	0.0622	ITK
ILMN_2767731	1.10	0.1509	ICOSL
ILMN_2631752	1.16	0.1986	CTLA4
ILMN_2589871	1.22	0.2097	CD28
ILMN_1225078	1.04	0.2288	ITK
ILMN_2743244	1.39	0.2296	CD86
ILMN_1252472	1.12	0.2446	ITK
ILMN_2906430	1.13	0.2563	ICOS
ILMN_1217041	1.28	0.2961	CD86
ILMN_1235456	1.05	0.3961	ICOSL
ILMN_1217899	0.99	0.9369	ITK
ILMN_1216386	1.00	0.9881	CD86
**IL7PATHWAY (NES = 2.04; FDR *q* = 0.0017)**			
**Probe ID**	**Fold**	***P*-value**	**Gene**
ILMN_2651137	2.23	0.0506	LCK
ILMN_1213278	1.24	0.0527	JAK1
ILMN_2976191	1.30	0.0565	STAT5A
ILMN_2800358	1.22	0.0609	EP300
ILMN_2625134	1.10	0.0656	EP300
ILMN_1230007	1.19	0.0730	PTK2B

ILMN_2680827	1.54	0.0804	IL7R
ILMN_1249021	1.22	0.0881	BCL2
ILMN_2568028	1.25	0.0929	IL2RG
ILMN_2701093	1.09	0.0955	JAK1
ILMN_2682162	1.19	0.1088	BCL2
ILMN_2886646	1.50	0.1171	IL2RG
ILMN_2552473	1.12	0.1434	JAK1
ILMN_2751207	1.16	0.1722	FYN
ILMN_2659739	1.16	0.2608	IL7R
ILMN_1219155	1.20	0.3013	JAK3
ILMN_2746204	1.04	0.3052	CREBBP
ILMN_2630852	1.16	0.3427	IL7
ILMN_2975500	0.96	0.3716	IL7R
ILMN_1213727	1.03	0.4045	IL7
ILMN_3006451	1.05	0.4451	JAK3
ILMN_2479839	0.98	0.4584	EP300
ILMN_1255457	1.02	0.4727	CREBBP
ILMN_1248546	0.97	0.4945	IL7
ILMN_1237567	1.02	0.6248	IL7R
ILMN_2912082	0.98	0.6772	FYN
ILMN_1255750	1.02	0.6848	IL7R
ILMN_2663821	0.98	0.7219	FYN
ILMN_2976188	0.99	0.7960	STAT5A
ILMN_2695020	1.01	0.8674	CREBBP
**HSA04620_TOLL_LIKE_RECEPTOR_SIGNALING_PATHWAY (NES = 1.44; FDR *q* = 0.11)**			
**Probe ID**	**Fold**	***P*-value**	**Gene**
ILMN_1231814	2.30	0.0282	CCL5
ILMN_3128616	1.72	0.0395	PIK3CD
ILMN_1243876	1.37	0.0563	CASP8
ILMN_2473531	0.89	0.0568	PIK3R1
ILMN_2642239	1.13	0.0733	NFKB2
ILMN_2614777	1.09	0.0868	MAPK11
ILMN_1236908	1.24	0.0878	TLR1
ILMN_2502136	1.47	0.0946	CD40
ILMN_2598703	1.14	0.1039	IFNAR2
ILMN_3115796	1.59	0.1090	CD40
ILMN_2592476	1.28	0.1107	NFKB1
ILMN_2564205	1.32	0.1170	PIK3CG
ILMN_1247446	1.07	0.1174	CXCL11
ILMN_2454579	1.04	0.1397	TLR9
ILMN_3097868	1.15	0.1440	CASP8
ILMN_2687005	1.06	0.1501	IFNA14
ILMN_1235547	1.06	0.1530	IKBKB
ILMN_2743003	1.22	0.1810	LY96
ILMN_1227086	1.22	0.1828	PIK3CG
ILMN_2621752	1.31	0.1848	IRF5
ILMN_1245918	0.93	0.1883	MAP3K7IP1
ILMN_2716622	1.23	0.2080	MAPK11

ILMN_2714534	1.04	0.2121	MAP2K1
ILMN_1225311	0.95	0.2165	IFNAR2
ILMN_3162337	1.21	0.2188	TLR6
ILMN_2743244	1.39	0.2296	CD86
ILMN_3161543	1.18	0.2301	IKBKE
ILMN_1218605	1.07	0.2317	NFKBIA
ILMN_2576736	1.23	0.2388	NFKB1
ILMN_2503129	1.09	0.2399	CD40
ILMN_2774646	1.08	0.2426	MYD88
ILMN_2439638	1.16	0.2472	TRAF3
ILMN_1223126	1.10	0.2486	FADD
ILMN_1226397	1.04	0.2505	IFNA14
ILMN_3066172	1.05	0.2643	TRAF3
ILMN_2721809	1.05	0.2713	MAPK14
ILMN_1238472	0.95	0.2828	IKBKB
ILMN_2768200	1.03	0.2860	MAP2K7
ILMN_2845312	0.79	0.2865	IFNA6
ILMN_3114641	0.94	0.2958	PIK3R1
ILMN_1217041	1.28	0.2961	CD86
ILMN_2769622	1.05	0.2991	IFNA6
ILMN_2598585	1.08	0.3003	IKBKG
ILMN_2697615	1.07	0.3164	MAPK12
ILMN_2955020	1.09	0.3241	TICAM2
ILMN_2971767	1.14	0.3255	MYD88
ILMN_3144289	1.12	0.3419	TRAF3
ILMN_1259162	1.05	0.3500	TRAF6
ILMN_2664319	1.07	0.3708	IRF3
ILMN_1213935	1.03	0.3752	AKT1
ILMN_2427560	1.07	0.3897	TRAF6
ILMN_1246274	0.97	0.3909	TRAF6
ILMN_2593196	1.14	0.4198	STAT1
ILMN_2655721	1.20	0.4239	STAT1
ILMN_2589556	1.07	0.4331	IKBKB
ILMN_3028981	0.96	0.4371	MAP2K7
ILMN_3040050	1.04	0.4384	PIK3R1
ILMN_2658633	1.03	0.4409	IFNA7
ILMN_1215521	1.04	0.4416	MAP2K6
ILMN_3001914	1.13	0.4435	NFKBIA
ILMN_1233783	1.03	0.4437	MAP3K7IP1
ILMN_2510233	1.06	0.4698	STAT1
ILMN_1249335	0.97	0.4789	PIK3R1
ILMN_2662509	1.04	0.4840	FADD
ILMN_2812164	1.06	0.4919	IRAK4
ILMN_1248521	1.04	0.5072	PIK3R3
ILMN_1224472	1.07	0.5217	CCL4
ILMN_1253919	1.07	0.5294	CCL3
ILMN_2740859	1.07	0.5350	RELA
ILMN_2641897	1.02	0.5391	IFNA13
ILMN_1241535	1.07	0.5412	TBK1

ILMN_2904321	1.13	0.5473	AKT3
ILMN_1259290	1.04	0.5758	AKT3
ILMN_1229320	1.04	0.6013	IRF3
ILMN_2496288	1.02	0.6044	CD40
ILMN_2698097	0.98	0.6121	IKBKG
ILMN_3102163	1.03	0.6255	MAP2K7
ILMN_1230562	0.98	0.6325	IFNA6
ILMN_1239417	1.03	0.6559	TRAF6
ILMN_2672136	0.97	0.6597	IRAK4
ILMN_2630423	1.02	0.6743	IKBKG
ILMN_2599748	1.02	0.6781	MAP2K6
ILMN_2874409	0.98	0.7002	FADD
ILMN_1254605	1.02	0.7210	TLR6
ILMN_2665625	0.98	0.7213	FADD
ILMN_1224505	0.97	0.7395	IRF3
ILMN_3052174	0.98	0.7438	PIK3CD
ILMN_2655112	1.01	0.7852	PIK3CD
ILMN_2906042	1.01	0.8108	MAP3K7IP1
ILMN_1248106	0.99	0.8192	MAPK12
ILMN_1223257	1.04	0.8231	CCL4
ILMN_2960750	1.01	0.8324	TRAF6
ILMN_2971514	0.98	0.8679	IFNAR2
ILMN_1242716	1.00	0.8903	IFNA6
ILMN_1226157	1.02	0.8906	PIK3R3
ILMN_2731482	1.00	0.9131	MAP3K7IP1
ILMN_2623856	1.00	0.9252	MAP2K6
ILMN_3035785	1.00	0.9329	MAP2K7
ILMN_1226783	1.00	0.9596	IRAK4
ILMN_2849322	1.00	0.9621	TLR9
ILMN_2724570	1.00	0.9869	MAPK12
ILMN_1216386	1.00	0.9881	CD86
**KREBS_TCA_CYCLE (NES = 1.85; FDR *q* = 0.0094)**			
**Probe ID**	**Fold**	***P*-value**	**Gene**
ILMN_2873112	1.17	0.0027	MDH2
ILMN_1220173	1.09	0.0139	PPM2C
ILMN_1239143	1.13	0.0343	SDHD
ILMN_2631021	1.07	0.0764	MDH1
ILMN_2691697	1.06	0.0938	OGDH
ILMN_2676876	1.13	0.1164	PDK3
ILMN_1237507	1.11	0.1702	DLST
ILMN_2564352	1.05	0.1770	SDHA
ILMN_1241236	1.09	0.2003	CS
ILMN_2703657	1.04	0.2152	DLD
ILMN_1260206	0.97	0.3775	DLD
ILMN_2655677	1.03	0.4609	MDH1
ILMN_2891176	0.97	0.4787	MDH1
ILMN_1240450	0.98	0.4964	SDHA
ILMN_2692509	1.02	0.7452	DLST

ILMN_2639805	0.98	0.7954	OGDH
ILMN_3162452	1.00	0.9541	PPM2C
