# Supplementary material for: Metabolic Regulation in Progression to Autoimmune Diabetes
Source: PLoS Comput Biol. 2011 Oct 27;7(10):e1002257. doi: 10.1371/journal.pcbi.1002257 (PMC3203065; doi:10.1371/journal.pcbi.1002257)
Supplement: Table S3 — Pathway analysis of liver transcriptomics data. Gene Set Enrichment Analysis [20] results at FDR q<0.25 for three different comparisons: (1) High diabetes risk (HR) vs. low risk (LR); (2) IAA+ vs. IAA−; (3) IAA+ LR vs. other. Transcriptomics was performed on n = 12 19-week old female NOD mice (four IAA+ LR, three IAA− LR, two IAA+ HR, three IAA− HR). N, number of genes in the pathway; NES, normalized enrichment score; FDR q, False Discovery Rate q-value [15]; Source, gene list source. (PDF) [file pcbi.1002257.s006.pdf]

Progressors *vs*. non-progressors				
**Pathway**	**N**	**NES**	**FDR *q***	**Source**
EDG1PATHWAY	22	1.80	0.074110	BioCarta
ERKPATHWAY	28	1.84	0.077616	BioCarta
HSA00670_ONE_CARBON_POOL_BY_FOLATE	15	1.75	0.084030	KEGG
HSA00563_GLYCOSYLPHOSPHATIDYLINOSITOL_ANCHOR_BIOSYNTHESIS	21	1.86	0.084170	KEGG
CELL_MOTILITY	90	1.75	0.084275	GO
HSA04115_P53_SIGNALING_PATHWAY	62	1.76	0.084678	KEGG
ATMPATHWAY	17	1.75	0.084693	BioCarta
HSA03022_BASAL_TRANSCRIPTION_FACTORS	30	1.74	0.085356	KEGG
HSA05216_THYROID_CANCER	26	1.75	0.086987	KEGG
HSA04360_AXON_GUIDANCE	122	1.74	0.087133	KEGG
HSA04530_TIGHT_JUNCTION	116	1.77	0.087402	KEGG
HSA00626_NAPHTHALENE_AND_ANTHRACENE_DEGRADATION	17	1.74	0.087777	KEGG
NUCLEAR_RECEPTORS	38	1.77	0.087822	GenMAPP
RNA_TRANSCRIPTION_REACTOME	34	1.70	0.094451	GenMAPP
BIOPEPTIDESPATHWAY	36	1.64	0.119179	BioCarta
HSA01031_GLYCAN_STRUCTURES_BIOSYNTHESIS_2	56	1.64	0.119692	KEGG
HSA04910_INSULIN_SIGNALING_PATHWAY	124	1.62	0.120935	KEGG
SA_PTEN_PATHWAY	16	1.64	0.121073	SigmaAldrich
HSA00440_AMINOPHOSPHONATE_METABOLISM	15	1.65	0.121642	KEGG
ATRBRCAPATHWAY	18	1.63	0.121746	BioCarta
GLUTATHIONE_METABOLISM	30	1.62	0.123294	GenMAPP
NFKBPATHWAY	23	1.60	0.124794	BioCarta
ECMPATHWAY	20	1.63	0.125733	BioCarta
TIDPATHWAY	16	1.61	0.125794	BioCarta
GO_ROS	27	1.61	0.125859	GO
FMLPPATHWAY	36	1.63	0.126087	BioCarta
WNT_SIGNALING	58	1.63	0.126643	GEArray
OVARIAN_INFERTILITY_GENES	25	1.59	0.127367	GenMAPP
HSA05214_GLIOMA	58	1.59	0.128191	KEGG
HSA04510_FOCAL_ADHESION	185	1.58	0.128434	KEGG
HSA04920_ADIPOCYTOKINE_SIGNALING_PATHWAY	71	1.58	0.128910	KEGG
HSA01510_NEURODEGENERATIVE_DISEASES	36	1.59	0.129177	KEGG
HSA05219_BLADDER_CANCER	37	1.57	0.129643	KEGG
HSA00770_PANTOTHENATE_AND_COA_BIOSYNTHESIS	15	1.57	0.130170	KEGG
HSA04662_B_CELL_RECEPTOR_SIGNALING_PATHWAY	59	1.57	0.131478	KEGG
INTEGRIN_MEDIATED_CELL_ADHESION_KEGG	90	1.57	0.132717	GenMAPP
MRNA_PROCESSING_REACTOME	102	1.56	0.134787	GenMAPP
HSA00260_GLYCINE_SERINE_AND_THREONINE_METABOLISM	43	1.55	0.141282	KEGG
HSA00450_SELENOAMINO_ACID_METABOLISM	25	1.55	0.142997	KEGG
HSA03050_PROTEASOME	22	1.55	0.144098	KEGG
HSA04310_WNT_SIGNALING_PATHWAY	134	1.54	0.144522	KEGG
ARFPATHWAY	15	1.53	0.148120	BioCarta
ST_T_CELL_SIGNAL_TRANSDUCTION	44	1.54	0.148424	STKE
HSA05020_PARKINSONS_DISEASE	15	1.52	0.152607	KEGG
STATIN_PATHWAY_PHARMGKB	17	1.52	0.157357	GenMAPP
SIG_CHEMOTAXIS	44	1.52	0.158416	SignalingAlliance
HSA04916_MELANOGENESIS	92	1.51	0.163552	KEGG

HSA05220_CHRONIC_MYELOID_LEUKEMIA	71	1.51	0.164393	KEGG
HSA00010_GLYCOLYSIS_AND_GLUCONEOGENESIS	53	1.51	0.164446	KEGG
CXCR4PATHWAY	22	1.50	0.167945	BioCarta
HSA04730_LONG_TERM_DEPRESSION	70	1.50	0.168420	KEGG
NO2IL12PATHWAY	15	1.50	0.169179	BioCarta
IL1RPATHWAY	32	1.50	0.169621	BioCarta
GLYCOSPHINGOLIPID_METABOLISM	19	1.49	0.174203	GenMAPP
G1_TO_S_CELL_CYCLE_REACTOME	65	1.49	0.174421	GenMAPP
HSA00480_GLUTATHIONE_METABOLISM	34	1.49	0.175293	KEGG
PTDINSPATHWAY	21	1.49	0.175595	BioCarta
TYPE_III_SECRETION_SYSTEM	19	1.49	0.175650	GenMAPP
HSA04370_VEGF_SIGNALING_PATHWAY	65	1.49	0.176019	KEGG
HSA04020_CALCIUM_SIGNALING_PATHWAY	161	1.48	0.176726	KEGG
FLAGELLAR_ASSEMBLY	19	1.47	0.179115	GenMAPP
HSA04630_JAK_STAT_SIGNALING_PATHWAY	138	1.47	0.179526	KEGG
HSA00150_ANDROGEN_AND_ESTROGEN_METABOLISM	37	1.47	0.179693	KEGG
HSA04720_LONG_TERM_POTENTIATION	62	1.47	0.179707	KEGG
CELL_CYCLE_ARREST	26	1.47	0.179779	GO
RIBOSOMAL_PROTEINS	71	1.47	0.180099	GenMAPP
GLYCINE_SERINE_AND_THREONINE_METABOLISM	34	1.47	0.180134	GenMAPP
ST_ERK1_ERK2_MAPK_PATHWAY	29	1.47	0.180533	STKE
PROTEASOME	17	1.47	0.180617	GenMAPP
ATP_SYNTHESIS	19	1.48	0.180859	GenMAPP
CELL_CYCLE	71	1.46	0.181256	GO
P53HYPOXIAPATHWAY	17	1.45	0.181575	BioCarta
HSA04512_ECM_RECEPTOR_INTERACTION	83	1.45	0.181622	KEGG
PHOTOSYNTHESIS	20	1.46	0.181815	GenMAPP
CHEMICALPATHWAY	19	1.46	0.181833	BioCarta
HSA00860_PORPHYRIN_AND_CHLOROPHYLL_METABOLISM	29	1.48	0.181956	KEGG
COMPPATHWAY	15	1.46	0.182160	BioCarta
PTENPATHWAY	16	1.45	0.182665	BioCarta
HYPERTROPHY_MODEL	16	1.45	0.182746	GenMAPP
ST_FAS_SIGNALING_PATHWAY	58	1.45	0.182910	STKE
HSA00350_TYROSINE_METABOLISM	53	1.46	0.183566	KEGG
HSA00980_METABOLISM_OF_XENOBIOTICS_BY_CYTOCHROME_P450	39	1.46	0.183650	KEGG
HSA00533_KERATAN_SULFATE_BIOSYNTHESIS	15	1.44	0.190798	KEGG
SIG_REGULATION_OF_THE_ACTIN_CYTOSKELETON_BY_RHO_GTPASES	34	1.44	0.190836	SignalingAlliance
HSA04660_T_CELL_RECEPTOR_SIGNALING_PATHWAY	90	1.43	0.193678	KEGG
HSA04940_TYPE_I_DIABETES_MELLITUS	21	1.43	0.194057	KEGG
HIVNEFPATHWAY	53	1.43	0.194440	BioCarta
ST_GAQ_PATHWAY	26	1.43	0.194625	STKE
ST_GA13_PATHWAY	33	1.43	0.195504	STKE
CELL_CYCLE_KEGG	79	1.42	0.199641	GenMAPP
HSA04520_ADHERENS_JUNCTION	70	1.42	0.200074	KEGG
IGF1MTORPATHWAY	19	1.42	0.201500	BioCarta
HDACPATHWAY	28	1.42	0.203771	BioCarta
DNA_REPLICATION_REACTOME	40	1.41	0.206845	GenMAPP
ST_P38_MAPK_PATHWAY	36	1.41	0.208067	STKE
HSA05221_ACUTE_MYELOID_LEUKEMIA	50	1.40	0.216068	KEGG

CELL_GROWTH_AND_OR_MAINTENANCE	58	1.40	0.216729	GO
HSA00510_N_GLYCAN_BIOSYNTHESIS	40	1.40	0.219178	KEGG
HSA03020_RNA_POLYMERASE	19	1.40	0.219499	KEGG
SIG_PIP3_SIGNALING_IN_CARDIAC_MYOCTES	63	1.40	0.219756	SignalingAlliance
CALCIUM_REGULATION_IN_CARDIAC_CELLS	135	1.40	0.220132	GenMAPP
EIF4PATHWAY	22	1.40	0.220705	BioCarta
HSA00052_GALACTOSE_METABOLISM	28	1.40	0.220719	KEGG
HSA04210_APOPTOSIS	75	1.39	0.221115	KEGG
HSA05120_EPITHELIAL_CELL_SIGNALING_IN_HELICOBACTER_PYLORI_INFECTION	64	1.39	0.223214	KEGG
ST_DICTYOSTELIUM_DISCOIDEUM_CAMP_CHEMOTAXIS_PATHWAY	31	1.39	0.223255	STKE
BUTANOATE_METABOLISM	26	1.39	0.223623	GenMAPP
G_PROTEIN_SIGNALING	85	1.39	0.223806	GenMAPP
HSA04930_TYPE_II_DIABETES_MELLITUS	42	1.38	0.224568	KEGG
HSA05222_SMALL_CELL_LUNG_CANCER	83	1.38	0.224993	KEGG
HSA05213_ENDOMETRIAL_CANCER	49	1.38	0.225753	KEGG
OXIDATIVE_PHOSPHORYLATION	56	1.38	0.226946	GenMAPP
HSA00790_FOLATE_BIOSYNTHESIS	37	1.38	0.227902	KEGG
MPRPATHWAY	20	1.37	0.228233	BioCarta
GALACTOSE_METABOLISM	21	1.38	0.228233	GenMAPP
APOPTOSIS_KEGG	46	1.37	0.228485	GenMAPP
ALANINE_AND_ASPARTATE_METABOLISM	18	1.36	0.232958	GenMAPP
HSA04010_MAPK_SIGNALING_PATHWAY	240	1.37	0.233813	KEGG
HSA04330_NOTCH_SIGNALING_PATHWAY	40	1.36	0.234705	KEGG
ST_B_CELL_ANTIGEN_RECEPTOR	37	1.36	0.235038	STKE
GCRPATHWAY	17	1.36	0.235589	BioCarta
GLUCONEOGENESIS	45	1.36	0.236091	GenMAPP
HSA00500_STARCH_AND_SUCROSE_METABOLISM	62	1.35	0.236200	KEGG
HSA04540_GAP_JUNCTION	81	1.35	0.236292	KEGG
HSA04742_TASTE_TRANSDUCTION	27	1.35	0.239806	KEGG
GLYCOLYSIS	45	1.35	0.241468	GenMAPP
SIG_CD40PATHWAYMAP	33	1.35	0.242235	SignalingAlliance
HSA04012_ERBB_SIGNALING_PATHWAY	81	1.34	0.245151	KEGG
BIOGENIC_AMINE_SYNTHESIS	15	1.34	0.245608	GenMAPP
ST_DIFFERENTIATION_PATHWAY_IN_PC12_CELLS	40	1.34	0.246162	STKE
CCR3PATHWAY	20	1.34	0.249484	BioCarta
**IAA+ *vs.* IAA-**				
**Pathway**	**N**	**NES**	**FDR *q***	**Source**
HSA04620_TOLL_LIKE_RECEPTOR_SIGNALING_PATHWAY	96	2.19	0.000421	KEGG
HSA05130_PATHOGENIC_ESCHERICHIA_COLI_INFECTION_EHEC	41	2.18	0.000459	KEGG
HSA00531_GLYCOSAMINOGLYCAN_DEGRADATION	15	2.19	0.000473	KEGG
HSA05131_PATHOGENIC_ESCHERICHIA_COLI_INFECTION_EPEC	41	2.17	0.000526	KEGG
APOPTOSIS	63	2.15	0.000637	GenMAPP
HSA04110_CELL_CYCLE	106	1.99	0.003305	KEGG
HSA04520_ADHERENS_JUNCTION	70	1.95	0.004542	KEGG
CELL_CYCLE_KEGG	79	1.93	0.005582	GenMAPP
ST_GRANULE_CELL_SURVIVAL_PATHWAY	25	1.90	0.006987	STKE
HSA04612_ANTIGEN_PROCESSING_AND_PRESENTATION	42	1.90	0.007031	KEGG
NFKBPATHWAY	23	1.89	0.007321	BioCarta

TCRPATHWAY	41	1.89	0.007360	BioCarta
IL7PATHWAY	16	1.88	0.007970	BioCarta
HSA05212_PANCREATIC_CANCER	71	1.87	0.008455	KEGG
STRIATED_MUSCLE_CONTRACTION	32	1.86	0.008867	GenMAPP
COMPPATHWAY	15	1.84	0.009617	BioCarta
HSA05214_GLIOMA	58	1.84	0.009623	KEGG
ST_MYOCYTE_AD_PATHWAY	23	1.84	0.009680	STKE
FCER1PATHWAY	36	1.85	0.009781	BioCarta
PHOSPHATIDYLINOSITOL_SIGNALING_SYSTEM	83	1.83	0.010348	GenMAPP
METPATHWAY	34	1.83	0.010368	BioCarta
NTHIPATHWAY	21	1.83	0.010389	BioCarta
HSA04650_NATURAL_KILLER_CELL_MEDIATED_CYTOTOXICITY	94	1.83	0.010510	KEGG
HSA04740_OLFACTORY_TRANSDUCTION	27	1.81	0.011686	KEGG
HSA05218_MELANOMA	68	1.80	0.012261	KEGG
RELAPATHWAY	16	1.80	0.012274	BioCarta
HSA05010_ALZHEIMERS_DISEASE	26	1.80	0.012304	KEGG
CTLA4PATHWAY	16	1.79	0.012472	BioCarta
G1_TO_S_CELL_CYCLE_REACTOME	65	1.79	0.012589	GenMAPP
HSA04720_LONG_TERM_POTENTIATION	62	1.79	0.012625	KEGG
IL12PATHWAY	20	1.79	0.013132	BioCarta
SIG_CHEMOTAXIS	44	1.78	0.013546	SignalingAlliance
APOPTOSIS_GENMAPP	40	1.78	0.013818	GenMAPP
SIG_PIP3_SIGNALING_IN_B_LYMPHOCYTES	33	1.77	0.013867	SignalingAlliance
TNFR2PATHWAY	18	1.78	0.013869	BioCarta
ST_DIFFERENTIATION_PATHWAY_IN_PC12_CELLS	40	1.77	0.014002	STKE
TOLLPATHWAY	34	1.76	0.014525	BioCarta
CALCINEURIN_NF_AT_SIGNALING	86	1.76	0.014588	GEArray
CELL_CYCLE	71	1.76	0.014669	GO
ETSPATHWAY	15	1.76	0.014835	BioCarta
HSA04662_B_CELL_RECEPTOR_SIGNALING_PATHWAY	59	1.76	0.014843	KEGG
HSA04810_REGULATION_OF_ACTIN_CYTOSKELETON	193	1.76	0.015055	KEGG
STRESSPATHWAY	25	1.75	0.016324	BioCarta
HSA05215_PROSTATE_CANCER	82	1.74	0.016627	KEGG
HSA04660_T_CELL_RECEPTOR_SIGNALING_PATHWAY	90	1.74	0.017209	KEGG
NO2IL12PATHWAY	15	1.74	0.017589	BioCarta
HSA00534_HEPARAN_SULFATE_BIOSYNTHESIS	18	1.73	0.017594	KEGG
HSA04210_APOPTOSIS	75	1.73	0.017632	KEGG
CALCINEURINPATHWAY	18	1.73	0.018253	BioCarta
ST_ADRENERGIC	31	1.73	0.018404	STKE
ST_INTEGRIN_SIGNALING_PATHWAY	78	1.73	0.018767	STKE
CELL_CYCLE_CHECKPOINT	22	1.72	0.018896	GO
GLEEVECPATHWAY	21	1.72	0.018938	BioCarta
MEF2DPATHWAY	17	1.72	0.019384	BioCarta
HSA04120_UBIQUITIN_MEDIATED_PROTEOLYSIS	36	1.72	0.019515	KEGG
ST_FAS_SIGNALING_PATHWAY	58	1.71	0.019905	STKE
HSA01032_GLYCAN_STRUCTURES_DEGRADATION	27	1.71	0.020303	KEGG
PROTEASOME	17	1.70	0.020714	GenMAPP
SIG_CD40PATHWAYMAP	33	1.70	0.021521	SignalingAlliance
HIVNEFPATHWAY	53	1.70	0.021941	BioCarta

CCR5PATHWAY	17	1.69	0.022924	BioCarta
PDGFPATHWAY	25	1.69	0.022977	BioCarta
TNFR1PATHWAY	27	1.68	0.024966	BioCarta
HSA05220_CHRONIC_MYELOID_LEUKEMIA	71	1.68	0.025430	KEGG
HSA05210_COLORECTAL_CANCER	81	1.68	0.025436	KEGG
BREAST_CANCER_ESTROGEN_SIGNALING	83	1.67	0.026681	GEArray
G_PROTEIN_SIGNALING	85	1.66	0.028286	GenMAPP
HSA04012_ERBB_SIGNALING_PATHWAY	81	1.65	0.029177	KEGG
ST_GA13_PATHWAY	33	1.65	0.029604	STKE
SIG_BCR_SIGNALING_PATHWAY	46	1.65	0.029616	SignalingAlliance
APOPTOSIS_KEGG	46	1.64	0.031388	GenMAPP
PORPHYRIN_AND_CHLOROPHYLL_METABOLISM	16	1.63	0.033081	GenMAPP
SMOOTH_MUSCLE_CONTRACTION	137	1.63	0.033227	GenMAPP
CARM_ERPATHWAY	24	1.62	0.035501	BioCarta
PROSTAGLANDIN_SYNTHESIS_REGULATION	28	1.62	0.036192	GenMAPP
CELLCYCLEPATHWAY	22	1.61	0.037349	BioCarta
HSA04510_FOCAL_ADHESION	185	1.61	0.037914	KEGG
ST_JNK_MAPK_PATHWAY	38	1.61	0.038027	STKE
DEATHPATHWAY	30	1.61	0.038606	BioCarta
ST_TUMOR_NECROSIS_FACTOR_PATHWAY	29	1.60	0.040363	STKE
HSA04540_GAP_JUNCTION	81	1.60	0.040372	KEGG
EGFPATHWAY	25	1.59	0.041361	BioCarta
KERATINOCYTEPATHWAY	42	1.59	0.041443	BioCarta
INTEGRIN_MEDIATED_CELL_ADHESION_KEGG	90	1.59	0.044131	GenMAPP
PGC1APATHWAY	22	1.58	0.044400	BioCarta
TIDPATHWAY	16	1.58	0.044692	BioCarta
HSA05216_THYROID_CANCER	26	1.58	0.045415	KEGG
HSA05213_ENDOMETRIAL_CANCER	49	1.58	0.045544	KEGG
HSA04010_MAPK_SIGNALING_PATHWAY	240	1.57	0.047805	KEGG
CELL_MOTILITY	90	1.57	0.048928	GO
CELL_ADHESION_RECEPTOR_ACTIVITY	32	1.57	0.049426	GO
HSA04610_COMPLEMENT_AND_COAGULATION_CASCADES	62	1.56	0.050775	KEGG
CELL_PROLIFERATION	187	1.56	0.050859	GO
NKCELLSPATHWAY	18	1.56	0.051679	BioCarta
GPCRPATHWAY	32	1.55	0.053067	BioCarta
DNA_REPLICATION_REACTOME	40	1.55	0.054461	GenMAPP
RNA_TRANSCRIPTION_REACTOME	34	1.55	0.055224	GenMAPP
HSA04940_TYPE_I_DIABETES_MELLITUS	21	1.55	0.055264	KEGG
HSA05223_NON_SMALL_CELL_LUNG_CANCER	50	1.54	0.057073	KEGG
CELL_ADHESION	166	1.54	0.057613	GO
HSA05221_ACUTE_MYELOID_LEUKEMIA	50	1.54	0.057720	KEGG
HSA04060_CYTOKINE_CYTOKINE_RECEPTOR_INTERACTION	218	1.53	0.058440	KEGG
HSA05222_SMALL_CELL_LUNG_CANCER	83	1.53	0.058574	KEGG
CELL_SURFACE_RECEPTOR_LINKED_SIGNAL_TRANSDUCTION	114	1.53	0.058670	GO
HSA04070_PHOSPHATIDYLINOSITOL_SIGNALING_SYSTEM	72	1.53	0.059507	KEGG
RAC1PATHWAY	22	1.53	0.060479	BioCarta
ST_G_ALPHA_I_PATHWAY	34	1.53	0.061064	STKE
CASPASEPATHWAY	21	1.52	0.062223	BioCarta
HSA05211_RENAL_CELL_CARCINOMA	67	1.52	0.063556	KEGG

BCRPATHWAY	32	1.51	0.063852	BioCarta
NOS1PATHWAY	20	1.51	0.065363	BioCarta
HSA04360_AXON_GUIDANCE	122	1.51	0.066139	KEGG
ARFPATHWAY	15	1.51	0.066276	BioCarta
CREBPATHWAY	24	1.50	0.070039	BioCarta
FASPATHWAY	26	1.49	0.071560	BioCarta
CALCIUM_REGULATION_IN_CARDIAC_CELLS	135	1.49	0.072299	GenMAPP
ST_WNT_CA2_CYCLIC_GMP_PATHWAY	18	1.49	0.072413	STKE
PPARAPATHWAY	46	1.49	0.072416	BioCarta
GPCRS_CLASS_B_SECRETIN_LIKE	19	1.49	0.074460	GO
ATP_SYNTHESIS	19	-1.87	0.074862	GenMAPP
HSA04630_JAK_STAT_SIGNALING_PATHWAY	138	1.48	0.076373	KEGG
WNTPATHWAY	24	1.48	0.077094	BioCarta
HSA00670_ONE_CARBON_POOL_BY_FOLATE	15	1.48	0.078274	KEGG
GSK3PATHWAY	26	1.47	0.080564	BioCarta
MAPKPATHWAY	83	1.47	0.080932	BioCarta
HSA04350_TGF_BETA_SIGNALING_PATHWAY	84	1.47	0.081949	KEGG
MITOCHONDRIAPATHWAY	19	1.47	0.082266	BioCarta
GPCRDB_CLASS_B_SECRETIN_LIKE	19	1.47	0.082799	GenMAPP
HSA04514_CELL_ADHESION_MOLECULES	105	1.46	0.086727	KEGG
GPCRDB_OTHER	48	1.45	0.089394	GenMAPP
HSA00240_PYRIMIDINE_METABOLISM	82	1.45	0.090033	KEGG
HSA04310_WNT_SIGNALING_PATHWAY	134	1.44	0.091992	KEGG
ST_GAQ_PATHWAY	26	1.44	0.092143	STKE
PROTEASOMEPATHWAY	20	1.43	0.096886	BioCarta
TH1TH2PATHWAY	15	1.43	0.099300	BioCarta
HSA02010_ABC_TRANSPORTERS_GENERAL	41	1.43	0.099746	KEGG
TPOPATHWAY	20	1.43	0.100830	BioCarta
FLAGELLAR_ASSEMBLY	19	-1.88	0.101446	GenMAPP
HSA04670_LEUKOCYTE_TRANSENDOTHELIAL_MIGRATION	106	1.42	0.105064	KEGG
IL1RPATHWAY	32	1.41	0.106800	BioCarta
GLYCOLYSIS	45	1.41	0.106931	GenMAPP
HSA00271_METHIONINE_METABOLISM	16	1.41	0.110609	KEGG
MRNA_PROCESSING_REACTOME	102	1.40	0.112058	GenMAPP
GLUCONEOGENESIS	45	1.40	0.115109	GenMAPP
ERK5PATHWAY	15	1.40	0.116612	BioCarta
ST_T_CELL_SIGNAL_TRANSDUCTION	44	1.39	0.119521	STKE
HSA04916_MELANOGENESIS	92	1.39	0.120793	KEGG
HSA00010_GLYCOLYSIS_AND_GLUCONEOGENESIS	53	1.38	0.126037	KEGG
HSA04340_HEDGEHOG_SIGNALING_PATHWAY	54	1.38	0.127368	KEGG
FRUCTOSE_AND_MANNOSE_METABOLISM	23	1.38	0.127821	GenMAPP
HSA04930_TYPE_II_DIABETES_MELLITUS	42	1.37	0.130075	KEGG
HSA00230_PURINE_METABOLISM	135	1.37	0.131206	KEGG
HSA04664_FC_EPSILON_RI_SIGNALING_PATHWAY	73	1.37	0.132798	KEGG
CSKPATHWAY	18	1.37	0.132898	BioCarta
AMIPATHWAY	18	1.37	0.133067	BioCarta
HSA05219_BLADDER_CANCER	37	1.36	0.135124	KEGG
VIPPATHWAY	25	1.36	0.135369	BioCarta
GLYCEROLIPID_METABOLISM	37	1.36	0.135649	GenMAPP

CARDIACEGFPATHWAY	15	1.36	0.136884	BioCarta
WNT_SIGNALING	58	1.36	0.136966	GEArray
PHOTOSYNTHESIS	20	-1.76	0.137493	GenMAPP
HSA01510_NEURODEGENERATIVE_DISEASES	36	1.36	0.137917	KEGG
HSA05040_HUNTINGTONS_DISEASE	28	1.35	0.141470	KEGG
TYPE_III_SECRETION_SYSTEM	19	-1.92	0.142051	GenMAPP
INTEGRINPATHWAY	34	1.34	0.148508	BioCarta
SA_B_CELL_RECEPTOR_COMPLEXES	23	1.34	0.151296	SigmaAldrich
CARBON_FIXATION	18	1.34	0.152949	GenMAPP
ACTINYPATHWAY	18	1.33	0.155077	BioCarta
NKTPATHWAY	27	1.33	0.156727	BioCarta
HSA00640_PROPANOATE_METABOLISM	28	1.32	0.163094	KEGG
HCMVPATHWAY	16	1.32	0.163135	BioCarta
ST_ERK1_ERK2_MAPK_PATHWAY	29	1.32	0.168800	STKE
HSA04640_HEMATOPOIETIC_CELL_LINEAGE	65	1.31	0.171279	KEGG
HSA04614_RENIN_ANGIOTENSIN_SYSTEM	15	1.31	0.171973	KEGG
ATRBRCAPATHWAY	18	1.31	0.172140	BioCarta
HSA03050_PROTEASOME	22	1.31	0.172256	KEGG
IL2RBPATHWAY	35	1.31	0.175758	BioCarta
P53PATHWAY	15	1.30	0.178494	BioCarta
HSA00562_INOSITOL_PHOSPHATE_METABOLISM	48	1.30	0.182415	KEGG
ST_B_CELL_ANTIGEN_RECEPTOR	37	1.30	0.183784	STKE
HSA05110_CHOLERA_INFECTION	36	1.30	0.185588	KEGG
NGFPATHWAY	17	1.29	0.194353	BioCarta
CELL_CYCLE_REGULATOR	21	1.28	0.195612	GO
HSA04115_P53_SIGNALING_PATHWAY	62	1.28	0.197523	KEGG
HSA04912_GNRH_SIGNALING_PATHWAY	90	1.28	0.197771	KEGG
HYPERTROPHY_MODEL	16	1.28	0.198289	GenMAPP
P38MAPKPATHWAY	37	1.28	0.199303	BioCarta
HSA04020_CALCIUM_SIGNALING_PATHWAY	161	1.28	0.199756	KEGG
HSA00710_CARBON_FIXATION	20	1.28	0.200703	KEGG
PYRIMIDINE_METABOLISM	54	1.27	0.202976	GenMAPP
HSA00860_PORPHYRIN_AND_CHLOROPHYLL_METABOLISM	29	1.27	0.203787	KEGG
ANDROGEN_AND_ESTROGEN_METABOLISM	16	1.27	0.208541	GenMAPP
TOB1PATHWAY	16	1.26	0.214554	BioCarta
MCALPAINPATHWAY	22	1.24	0.234733	BioCarta
NO1PATHWAY	27	1.24	0.236059	BioCarta
INOSITOL_PHOSPHATE_METABOLISM	23	1.24	0.238345	GenMAPP
CELL_CYCLE_ARREST	26	1.24	0.238678	GO
VEGFPATHWAY	23	1.23	0.243169	BioCarta
INTRINSICPATHWAY	22	1.23	0.243817	BioCarta
AMINOACYL_TRNA_BIOSYNTHESIS	22	1.23	0.245737	GenMAPP
HDACPATHWAY	28	1.23	0.246262	BioCarta
**IAA+ non-progressors *vs.* other**				
**Pathway**	**N**	**NES**	**FDR *q***	**Source**
CALCINEURINPATHWAY	18	2.08	0.001399	BioCarta
FCER1PATHWAY	36	2.07	0.001627	BioCarta
HSA05130_PATHOGENIC_ESCHERICHIA_COLI_INFECTION_EHEC	41	2.02	0.001986	KEGG

GLEEVECPATHWAY	21	2.00	0.002548	BioCarta
PDGFPATHWAY	25	1.99	0.002824	BioCarta
TCRPATHWAY	41	1.98	0.002959	BioCarta
HSA05131_PATHOGENIC_ESCHERICHIA_COLI_INFECTION_EPEC	41	1.98	0.003019	KEGG
HSA00020_CITRATE_CYCLE	25	1.95	0.003960	KEGG
ST_GRANULE_CELL_SURVIVAL_PATHWAY	25	1.94	0.004022	STKE
EGFPATHWAY	25	1.93	0.004418	BioCarta
ST_DIFFERENTIATION_PATHWAY_IN_PC12_CELLS	40	1.93	0.004522	STKE
CITRATE_CYCLE_TCA_CYCLE	17	1.90	0.006488	GenMAPP
HSA04110_CELL_CYCLE	106	1.86	0.008299	KEGG
TOLLPATHWAY	34	1.87	0.008355	BioCarta
SIG_CD40PATHWAYMAP	33	1.85	0.008700	SignalingAlliance
PHOTOSYNTHESIS	20	-2.03	0.011036	GenMAPP
NOS1PATHWAY	20	1.83	0.011146	BioCarta
HSA04520_ADHERENS_JUNCTION	70	1.83	0.011832	KEGG
FLAGELLAR_ASSEMBLY	19	-2.04	0.013336	GenMAPP
GPCRPATHWAY	32	1.81	0.013688	BioCarta
NTHIPATHWAY	21	1.81	0.013693	BioCarta
SIG_CHEMOTAXIS	44	1.81	0.013695	SignalingAlliance
PROSTAGLANDIN_SYNTHESIS_REGULATION	28	1.81	0.013811	GenMAPP
BCRPATHWAY	32	1.79	0.015370	BioCarta
APOPTOSIS_GENMAPP	40	1.78	0.016681	GenMAPP
HSA04120_UBIQUITIN_MEDIATED_PROTEOLYSIS	36	1.78	0.016717	KEGG
TYPE_III_SECRETION_SYSTEM	19	-2.05	0.017246	GenMAPP
NFKBPATHWAY	23	1.77	0.018211	BioCarta
RELAPATHWAY	16	1.76	0.019065	BioCarta
HSA00534_HEPARAN_SULFATE_BIOSYNTHESIS	18	1.76	0.019290	KEGG
HSA04660_T_CELL_RECEPTOR_SIGNALING_PATHWAY	90	1.75	0.020793	KEGG
APOPTOSIS	63	1.75	0.020804	GenMAPP
HSA05210_COLORECTAL_CANCER	81	1.74	0.021347	KEGG
HSA00640_PROPANOATE_METABOLISM	28	1.74	0.021955	KEGG
HSA00252_ALANINE_AND_ASPARTATE_METABOLISM	30	1.74	0.022017	KEGG
HSA04630_JAK_STAT_SIGNALING_PATHWAY	138	1.72	0.024152	KEGG
KREBS_TCA_CYCLE	28	1.71	0.026436	GenMAPP
HSA04012_ERBB_SIGNALING_PATHWAY	81	1.71	0.026836	KEGG
HSA04210_APOPTOSIS	75	1.71	0.026955	KEGG
G1_TO_S_CELL_CYCLE_REACTOME	65	1.70	0.028099	GenMAPP
TPOPATHWAY	20	1.70	0.028388	BioCarta
INTRINSICPATHWAY	22	1.70	0.028504	BioCarta
PHOSPHATIDYLINOSITOL_SIGNALING_SYSTEM	83	1.70	0.028800	GenMAPP
HSA04510_FOCAL_ADHESION	185	1.69	0.029854	KEGG
ATP_SYNTHESIS	19	-2.07	0.030378	GenMAPP
ST_INTEGRIN_SIGNALING_PATHWAY	78	1.69	0.030702	STKE
HIVNEFPATHWAY	53	1.68	0.032276	BioCarta
STRIATED_MUSCLE_CONTRACTION	32	1.67	0.033759	GenMAPP
HSA04310_WNT_SIGNALING_PATHWAY	134	1.66	0.034390	KEGG
HSA04662_B_CELL_RECEPTOR_SIGNALING_PATHWAY	59	1.66	0.034570	KEGG
AMINOACYL_TRNA_BIOSYNTHESIS	22	1.66	0.034628	GenMAPP
HSA00120_BILE_ACID_BIOSYNTHESIS	30	1.66	0.034710	KEGG

HSA04350_TGF_BETA_SIGNALING_PATHWAY	84	1.66	0.034884	KEGG
HSA04130_SNARE_INTERACTIONS_IN_VESICULAR_TRANSPORT	30	1.66	0.034999	KEGG
HSA00710_CARBON_FIXATION	20	1.66	0.035067	KEGG
HSA05213_ENDOMETRIAL_CANCER	49	1.65	0.035226	KEGG
SA_B_CELL_RECEPTOR_COMPLEXES	23	1.65	0.035257	SigmaAldrich
IGF1PATHWAY	19	1.65	0.035588	BioCarta
CELL_CYCLE_KEGG	79	1.65	0.036747	GenMAPP
ST_WNT_CA2_CYCLIC_GMP_PATHWAY	18	1.64	0.036952	STKE
TNFR2PATHWAY	18	1.65	0.036967	BioCarta
VIPPATHWAY	25	1.64	0.037857	BioCarta
HSA05010_ALZHEIMERS_DISEASE	26	1.64	0.038316	KEGG
MRNA_PROCESSING_REACTOME	102	1.63	0.040083	GenMAPP
ST_ADRENERGIC	31	1.63	0.040472	STKE
CTLA4PATHWAY	16	1.62	0.043158	BioCarta
PGC1APATHWAY	22	1.62	0.044427	BioCarta
MEF2DPATHWAY	17	1.62	0.044693	BioCarta
HSA04720_LONG_TERM_POTENTIATION	62	1.61	0.045193	KEGG
ST_JNK_MAPK_PATHWAY	38	1.61	0.045456	STKE
RAC1PATHWAY	22	1.61	0.045606	BioCarta
HSA05214_GLIOMA	58	1.60	0.046556	KEGG
CCR5PATHWAY	17	1.60	0.047589	BioCarta
ST_INTERLEUKIN_4_PATHWAY	23	1.60	0.047792	STKE
INSULINPATHWAY	19	1.60	0.048562	BioCarta
CELL_CYCLE_CHECKPOINT	22	1.60	0.048825	GO
KERATINOCYTEPATHWAY	42	1.59	0.049587	BioCarta
HSA04620_TOLL_LIKE_RECEPTOR_SIGNALING_PATHWAY	96	1.59	0.050898	KEGG
HSA04140_REGULATION_OF_AUTOPHAGY	23	1.58	0.054573	KEGG
NFATPATHWAY	49	1.56	0.057455	BioCarta
IL7PATHWAY	16	1.56	0.058066	BioCarta
PPARAPATHWAY	46	1.56	0.058705	BioCarta
ANDROGEN_AND_ESTROGEN_METABOLISM	16	1.55	0.060472	GenMAPP
TIDPATHWAY	16	1.55	0.061144	BioCarta
IL12PATHWAY	20	1.55	0.061201	BioCarta
HSA05222_SMALL_CELL_LUNG_CANCER	83	1.55	0.061274	KEGG
CARBON_FIXATION	18	1.55	0.061403	GenMAPP
PTENPATHWAY	16	1.54	0.062341	BioCarta
GLUTAMATE_METABOLISM	22	1.54	0.062430	GenMAPP
SIG_PIP3_SIGNALING_IN_B_LYMPHOCYTES	33	1.54	0.062968	SignalingAlliance
HSA05212_PANCREATIC_CANCER	71	1.54	0.064775	KEGG
HSA00310_LYSINE_DEGRADATION	45	1.54	0.065005	KEGG
NGFPATHWAY	17	1.54	0.065090	BioCarta
DEATHPATHWAY	30	1.53	0.067115	BioCarta
METPATHWAY	34	1.53	0.067179	BioCarta
HSA05215_PROSTATE_CANCER	82	1.53	0.067435	KEGG
HSA04530_TIGHT_JUNCTION	116	1.53	0.067886	KEGG
HSA04060_CYTOKINE_CYTOKINE_RECEPTOR_INTERACTION	218	1.53	0.067940	KEGG
CELL_PROLIFERATION	187	1.52	0.069380	GO
COMPPATHWAY	15	1.52	0.069433	BioCarta
HSA04810_REGULATION_OF_ACTIN_CYTOSKELETON	193	1.52	0.070801	KEGG

STRESSPATHWAY	25	1.52	0.070899	BioCarta
ETSPATHWAY	15	1.52	0.070923	BioCarta
PROPANOATE_METABOLISM	28	1.52	0.071099	GenMAPP
HSA05223_NON_SMALL_CELL_LUNG_CANCER	50	1.51	0.071816	KEGG
HSA04610_COMPLEMENT_AND_COAGULATION_CASCADES	62	1.51	0.071873	KEGG
RNA_TRANSCRIPTION_REACTOME	34	1.51	0.071905	GenMAPP
CELL_MOTILITY	90	1.51	0.072594	GO
APOPTOSIS_KEGG	46	1.51	0.073164	GenMAPP
HSA04740_OLFACTORY_TRANSDUCTION	27	1.50	0.074772	KEGG
HSA05220_CHRONIC_MYELOID_LEUKEMIA	71	1.50	0.076683	KEGG
HSA03010_RIBOSOME	55	1.50	0.077033	KEGG
ALKPATHWAY	34	1.49	0.079445	BioCarta
NO2IL12PATHWAY	15	1.49	0.081064	BioCarta
FASPATHWAY	26	1.49	0.081213	BioCarta
NKCELLSPATHWAY	18	1.48	0.082145	BioCarta
CALCINEURIN_NF_AT_SIGNALING	86	1.47	0.086208	GEArray
HSA00251_GLUTAMATE_METABOLISM	27	1.47	0.087411	KEGG
CELL_ADHESION	166	1.47	0.088433	GO
MAPKPATHWAY	83	1.47	0.088769	BioCarta
41BBPATHWAY	18	1.46	0.092719	BioCarta
HSA00531_GLYCOSAMINOGLYCAN_DEGRADATION	15	1.45	0.094917	KEGG
CALCIUM_REGULATION_IN_CARDIAC_CELLS	135	1.44	0.098605	GenMAPP
ST_G_ALPHA_I_PATHWAY	34	1.44	0.101339	STKE
NO1PATHWAY	27	1.43	0.102958	BioCarta
CELL_CYCLE_ARREST	26	1.43	0.103098	GO
HSA04540_GAP_JUNCTION	81	1.43	0.104423	KEGG
GPCRDB_OTHER	48	1.43	0.105298	GenMAPP
SA_PTEN_PATHWAY	16	1.43	0.105385	SigmaAldrich
CARDIACEGFPATHWAY	15	1.42	0.107971	BioCarta
HSA05211_RENAL_CELL_CARCINOMA	67	1.42	0.110268	KEGG
HSA04010_MAPK_SIGNALING_PATHWAY	240	1.42	0.111468	KEGG
HSA05216_THYROID_CANCER	26	1.41	0.113080	KEGG
HSA04650_NATURAL_KILLER_CELL_MEDIATED_CYTOTOXICITY	94	1.41	0.113838	KEGG
CELLCYCLEPATHWAY	22	1.41	0.113903	BioCarta
BLOOD_CLOTTING_CASCADE	19	1.40	0.116970	GenMAPP
HSA04514_CELL_ADHESION_MOLECULES	105	1.40	0.118481	KEGG
BREAST_CANCER_ESTROGEN_SIGNALING	83	1.40	0.120969	GEArray
HSA01430_CELL_COMMUNICATION	119	1.40	0.121304	KEGG
HSA00240_PYRIMIDINE_METABOLISM	82	1.40	0.121387	KEGG
HSA00330_ARGININE_AND_PROLINE_METABOLISM	29	1.40	0.122970	KEGG
IL1RPATHWAY	32	1.40	0.123115	BioCarta
CELL_CYCLE_REGULATOR	21	1.39	0.126092	GO
HSA00230_PURINE_METABOLISM	135	1.38	0.127991	KEGG
HSA01510_NEURODEGENERATIVE_DISEASES	36	1.38	0.129151	KEGG
SIG_BCR_SIGNALING_PATHWAY	46	1.38	0.132890	SignalingAlliance
HDACPATHWAY	28	1.38	0.133045	BioCarta
CELL_ADHESION_RECEPTOR_ACTIVITY	32	1.38	0.133689	GO
CELL_CYCLE	71	1.37	0.135461	GO
HSA04115_P53_SIGNALING_PATHWAY	62	1.37	0.137942	KEGG

ALANINE_AND_ASPARTATE_METABOLISM	18	1.37	0.138013	GenMAPP
SA_TRKA_RECEPTOR	15	1.37	0.138621	SigmaAldrich
EIF4PATHWAY	22	1.37	0.140207	BioCarta
INTEGRIN_MEDIATED_CELL_ADHESION_KEGG	90	1.36	0.142898	GenMAPP
AT1RPATHWAY	30	1.36	0.147583	BioCarta
HSA04670_LEUKOCYTE_TRANSENDOTHELIAL_MIGRATION	106	1.36	0.147660	KEGG
ARFPATHWAY	15	1.36	0.148524	BioCarta
ST_MYOCYTE_AD_PATHWAY	23	1.36	0.148737	STKE
ST_GA13_PATHWAY	33	1.35	0.149304	STKE
HSA05221_ACUTE_MYELOID_LEUKEMIA	50	1.35	0.149966	KEGG
HSA00280_VALINE_LEUCINE_AND_ISOLEUCINE_DEGRADATION	40	1.35	0.153060	KEGG
G_PROTEIN_SIGNALING	85	1.35	0.153179	GenMAPP
HSA00051_FRUCTOSE_AND_MANNOSE_METABOLISM	37	1.35	0.153322	KEGG
TNFR1PATHWAY	27	1.34	0.154982	BioCarta
WNT_SIGNALING	58	1.34	0.155343	GEArray
HSA04916_MELANOGENESIS	92	1.34	0.155946	KEGG
VEGFPATHWAY	23	1.34	0.156158	BioCarta
CELL_SURFACE_RECEPTOR_LINKED_SIGNAL_TRANSDUCTION	114	1.34	0.159315	GO
TOB1PATHWAY	16	1.33	0.163444	BioCarta
HSA00510_N_GLYCAN_BIOSYNTHESIS	40	1.33	0.164884	KEGG
WNTPATHWAY	24	1.33	0.167589	BioCarta
P38MAPKPATHWAY	37	1.32	0.174768	BioCarta
SMOOTH_MUSCLE_CONTRACTION	137	1.31	0.177549	GenMAPP
DNA_REPLICATION_REACTOME	40	1.31	0.183768	GenMAPP
HSA00271_METHIONINE_METABOLISM	16	1.30	0.185456	KEGG
BILE_ACID_BIOSYNTHESIS	21	1.30	0.186820	GenMAPP
HSA05218_MELANOMA	68	1.29	0.200729	KEGG
HSA00970_AMINOACYL_TRNA_BIOSYNTHESIS	38	1.29	0.201813	KEGG
HSA04360_AXON_GUIDANCE	122	1.29	0.202694	KEGG
CARM_ERPATHWAY	24	1.28	0.203752	BioCarta
HSA01030_GLYCAN_STRUCTURES_BIOSYNTHESIS_1	103	1.28	0.205558	KEGG
INFLAMPATHWAY	26	1.28	0.209005	BioCarta
IL2RBPATHWAY	35	1.27	0.213967	BioCarta
HSA05217_BASAL_CELL_CARCINOMA	53	1.27	0.218841	KEGG
INTEGRINPATHWAY	34	1.27	0.219825	BioCarta
SIG_INSULIN_RECEPTOR_PATHWAY_IN_CARDIAC_MYOCYTES	49	1.26	0.223933	SignalingAlliance
FMLPPATHWAY	36	1.26	0.225116	BioCarta
MCALPAINPATHWAY	22	1.26	0.226763	BioCarta
SPRYPATHWAY	16	1.26	0.228120	BioCarta
MITOCHONDRIAL_FATTY_ACID_BETAOXIDATION	15	-1.58	0.230045	GenMAPP
HSA04020_CALCIUM_SIGNALING_PATHWAY	161	1.25	0.230060	KEGG
HSA05030_AMYOTROPHIC_LATERAL_SCLEROSIS	17	1.25	0.230342	KEGG
ST_DICTYOSTELIUM_DISCOIDEUM_CAMP_CHEMOTAXIS_PATHWAY	31	1.25	0.232157	STKE
CELL_GROWTH_AND_OR_MAINTENANCE	58	1.25	0.232186	GO
RIBOSOMAL_PROTEINS	71	1.25	0.233136	GenMAPP
HSA05219_BLADDER_CANCER	37	1.25	0.233787	KEGG
PURINE_METABOLISM	105	1.25	0.237111	GenMAPP
HSA00150_ANDROGEN_AND_ESTROGEN_METABOLISM	37	1.25	0.237290	KEGG
VALINE_LEUCINE_AND_ISOLEUCINE_DEGRADATION	34	1.24	0.238275	GenMAPP

ERK5PATHWAY	15	1.24	0.238328	BioCarta
HSA04930_TYPE_II_DIABETES_MELLITUS	42	1.24	0.239016	KEGG
HSA00590_ARACHIDONIC_ACID_METABOLISM	39	-1.63	0.246451	KEGG
